# Chiral cyclopentadienylruthenium sulfoxide catalysts for asymmetric redox bicycloisomerization

**DOI:** 10.3762/bjoc.12.110

**Published:** 2016-06-07

**Authors:** Barry M Trost, Michael C Ryan, Meera Rao

**Affiliations:** 1 Department of Chemistry, Stanford University, Stanford, California 94305-5580, USA

**Keywords:** asymmetric catalysis, [3.1.0] bicycles, [4.1.0] bicycles, cycloisomerization, 1,6-enyne, 1,7-enyne, ruthenium catalysis, sulfoxide

## Abstract

A full account of our efforts toward an asymmetric redox bicycloisomerization reaction is presented in this article. Cyclopentadienylruthenium (CpRu) complexes containing tethered chiral sulfoxides were synthesized via an oxidative [3 + 2] cycloaddition reaction between an alkyne and an allylruthenium complex. Sulfoxide complex **1** containing a *p*-anisole moiety on its sulfoxide proved to be the most efficient and selective catalyst for the asymmetric redox bicycloisomerization of 1,6- and 1,7-enynes. This complex was used to synthesize a broad array of [3.1.0] and [4.1.0] bicycles. Sulfonamide- and phosphoramidate-containing products could be deprotected under reducing conditions. Catalysis performed with enantiomerically enriched propargyl alcohols revealed a matched/mismatched effect that was strongly dependent on the nature of the solvent.

## Introduction

Due to their prevalence in natural products [1], medicinal targets [2], and materials [3], organic chemists have made the construction of cyclic, organic molecules one of the most important areas of research in their discipline. Of the available methods to affect cyclization, transition metal-catalyzed enyne cycloisomerizations [4] have been recognized as an atom- [5], step- [6], and redox-economical [7] class of reactions that are able to stitch together cyclic molecules quickly and efficiently.

**Scheme 1:**
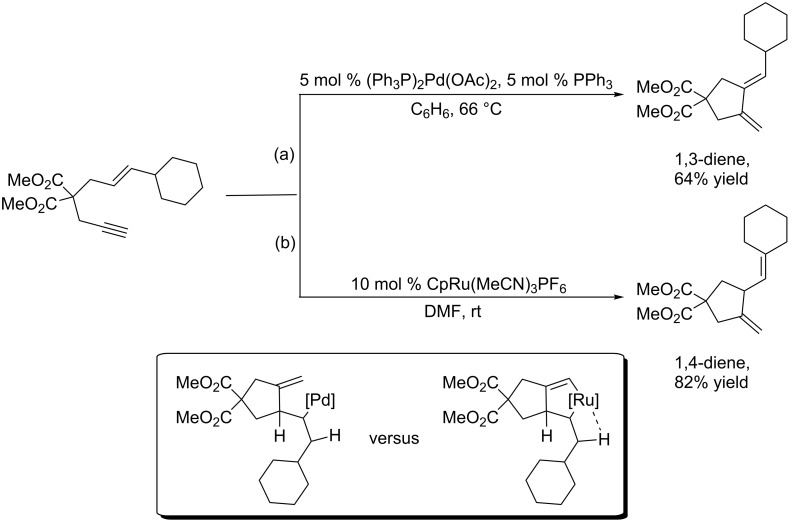
Divergent behavior of the palladium and ruthenium-catalyzed Alder–ene reaction*.*

The very first enyne cycloisomerization reactions were reported by the Trost group while they were synthesizing substrates intended for thermal Alder–ene reactions [8]. They serendipitously discovered that palladium(II) salts catalyzed the cyclization of 1,6-enynes at much lower temperatures compared to the thermal process [9], which normally requires temperatures in excess of 200 °C (Scheme 1, path a).

More recently, the same research group disclosed a CpRu(MeCN)_3_PF_6_-catalyzed variant of the same reaction that proceeds even at room temperature [10]. The ruthenium process differs from the initially-discovered palladium reaction in that it produces cyclic 1,4-dienes exclusively; no olefin isomerization is detected (Scheme 1, path b). Moreover, ruthenium can tolerate many sensitive functional groups, such as free alcohols, silyl enol ethers, and ketones, which makes it an attractive metal for late stage functionalization and elaboration of complex molecules. It is thought that the origin of ruthenium’s divergent behavior stems from a difference in reaction mechanism. Whereas the palladium-catalyzed Alder–ene reaction proceeds through an initial hydrometallation of a palladium hydride intermediate, ruthenium is speculated to first form a ruthenacyclopentene prior to β-hydride elimination. Since the hydrogen leading to the 1,3-diene is inaccessible to the ruthenacyclopentene, it must exclusively abstract the exocyclic hydrogen, which results in 1,4-diene formation. Palladium, which is not restricted to a metallacycle, is free to choose either hydrogen, and therefore performs β-hydride elimination on the allylic hydrogen.

**Scheme 2:**
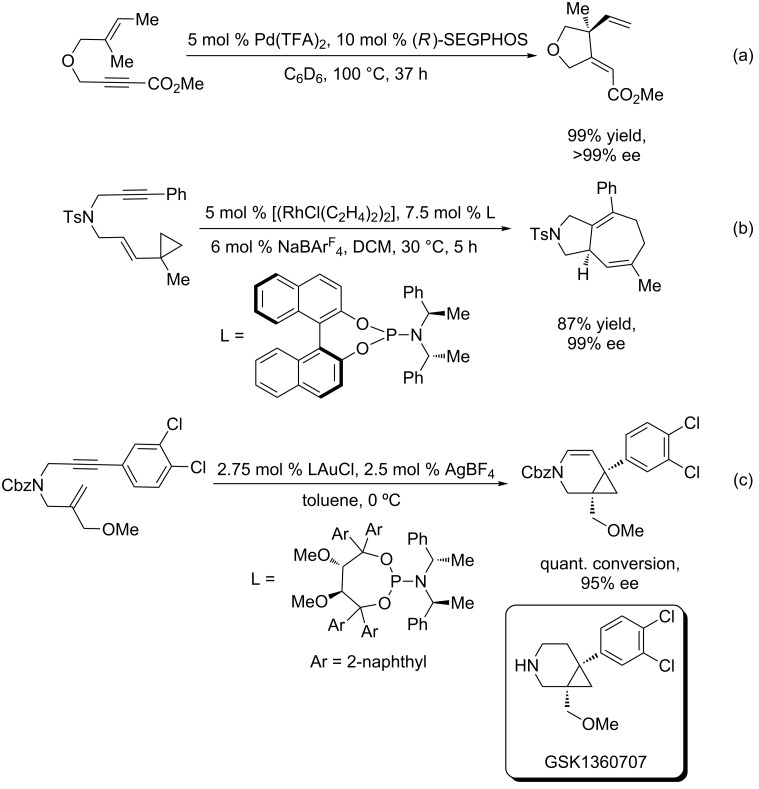
Some asymmetric enyne cycloisomerization reactions.

Recognizing that enantioenriched, cyclic molecular architectures hold a particular interest to the chemical community, especially in the fields of natural product synthesis and drug design, researchers have made a significant effort to discover asymmetric variations of enyne cycloisomerization reactions [11–12]. Researchers have used a variety of transition metals to affect asymmetric enyne cycloisomerization [13–23].^.^In particular, Mikami has disclosed a palladium-catalyzed asymmetric enyne cycloisomerization where a tetrahydrofuran containing a quaternary, all-carbon stereocenter is created in excellent yield and selectivity [24] (Scheme 2a). Unfortunately, the scope of this reaction is rather limited, as this is the only example presented in the paper. Mechanistic studies performed by the same group on this system support a hydropalladation/cyclization/β-hydride elimination mechanism. Rhodium catalysis of simple 1,6-enynes displayed a broader scope than Mikami’s palladium system, although none of the examples contained a quaternary stereocenter [25].

Asymmetric enyne cycloisomerization reactions can be extended beyond the construction of 1,4-dienes, depending on the transition metal used and the adjacent functionality on the substrate in question. For example, Hayashi has shown that a rhodium/phosphoramidite catalysis is particularly effective for asymmetric [5 + 2] cycloaddition reactions (Scheme 2b). The (*S*,*R*,*R)-*diastereomer of the Feringa-style phosphoramidite ligand proved to be crucial to both the yield and enantioselectivity of this reaction, as there was a severe matched/mismatched effect observed with another diastereomer. In contrast to the ruthenium-catalyzed [5 + 2] cycloaddition of enynes, which is thought to proceed through a ruthenacyclopentene intermediate [26], it has been proposed that rhodium first undergoes oxidative cyclization with the vinylcyclopropane prior to alkyne insertion.

The asymmetric enyne cycloisomerization reaction has been shown to be instrumental in the construction of medicinal chemistry targets. For example, the Fürstner group realized that gold catalysis would be particularly suited for the construction of the [4.1.0] bicyclic piperidine core of GSK1360707 [27], a triple-uptake inhibitor developed by GlaxoSmithKline [28–29] (Scheme 2c). The construction of this interesting molecular scaffold was a motivator of much of our early work on the ruthenium-catalyzed redox bicycloisomerization reaction (vide infra). Unlike ruthenium and palladium enyne cycloisomerization, which operate via metallacycle formation or hydrometallation, gold acts as a π-acid, increasing the electrophilicity of the alkyne by η^2^ coordination. The pendant alkene cyclizes on the alkyne, and the resulting tertiary carbocation is trapped by a gold carbenoid intermediate to form the fused cyclopropane.

**Figure 1:**
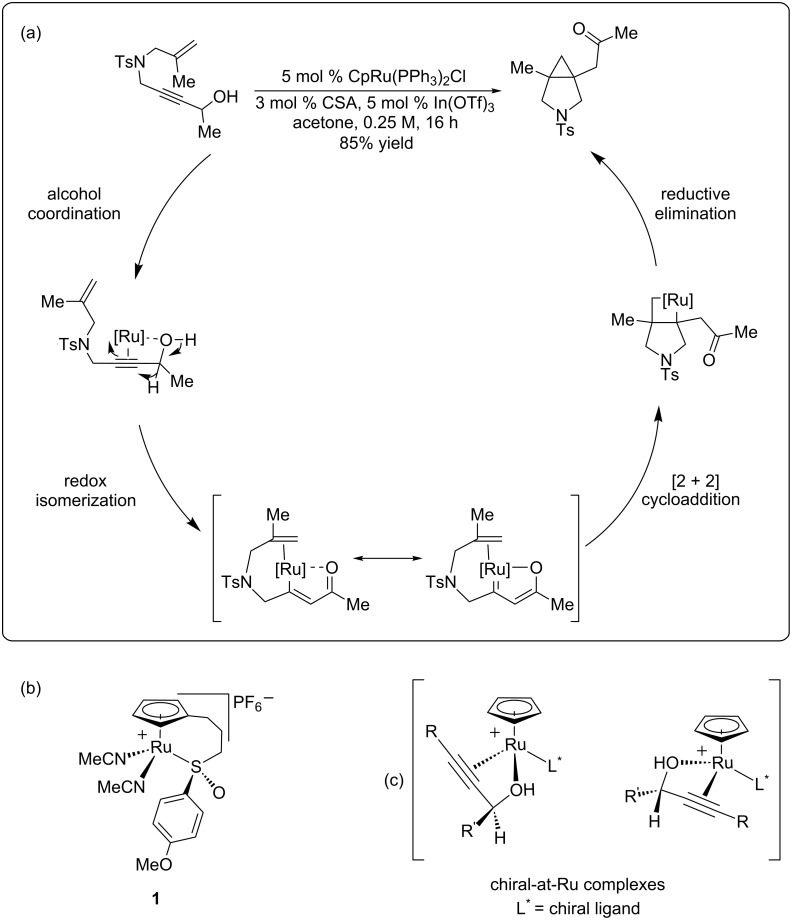
(a) Mechanism for the redox biscycloisomerization reaction. (b) Ruthenium catalyst containing a tethered chiral sulfoxide. (c) Possible diastereomeric complexes formed from alcohol coordination.

While there had been reports of utilizing chiral ruthenium complexes for asymmetric catalysis prior to our studies [30–40], there had previously been no reported examples of asymmetric ruthenium-catalyzed cycloisomerization reactions in the literature. In 2011, our research group disclosed the ruthenium-catalyzed redox bicycloisomerization of 1,6- and 1,7-enynes to construct structurally complex [3.1.0] and [4.1.0] bicycles containing vicinal, quaternary all-carbon stereocenters [41] (Figure 1a). The proposed mechanism of this fascinating reaction involves chloride abstraction of the ruthenium catalyst by indium(III) triflate and phosphine ligand dissociation. The propargyl alcohol then coordinates to the now coordinatively unsaturated cyclopentadienylruthenium (CpRu) fragment in a bidentate fashion and undergoes a redox isomerization reaction wherein the carbinol proton performs a 1,2-hydride shift. The resulting vinylruthenium intermediate can be seen as a resonance structure of a ruthenium carbene, which coordinates to a pendant alkene, performs a [2 + 2] cycloaddition to form a ruthenacyclobutane, and reductively eliminates to generate the bicyclic product.

Intrigued by the possibility of rendering this reaction asymmetric, we wondered if an appropriate choice of catalyst, namely the chiral CpRu-sulfoxide catalysts **1** our group developed for asymmetric allylic substitution reactions [42] (Figure 1b), would be able to impart sufficiently useful enantioselectivities on these complex, drug-like molecules. While the idea certainly was appealing at first glance, this reaction is complicated by the fact that the 1,6- and 1,7-enyne substrates contain a stereogenic center that, upon coordination to ruthenium, create diastereomeric, chiral-at-ruthenium complexes (Figure 1c). It was unclear a priori whether this transfer of stereochemical information would have an adventitious, negligible, or detrimental impact on the enantioselectivity of the reaction. With this important consideration in mind, we began our search for an asymmetric ruthenium-catalyzed redox bicycloisomerization reaction [43].

## Results and Discussion

### Catalyst synthesis

**Scheme 3:**
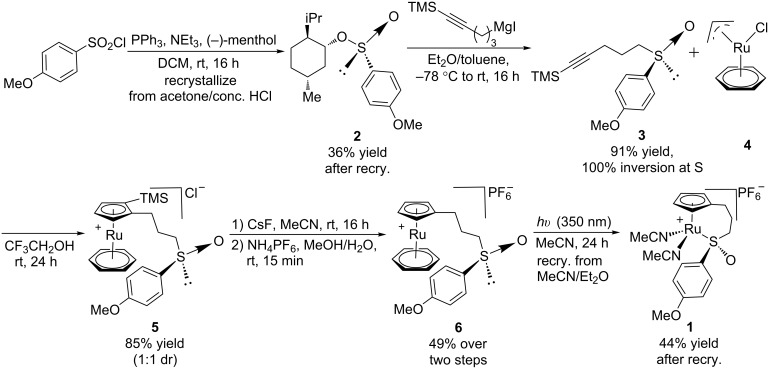
Synthesis of *p*-anisyl catalyst **1**.

Synthesis of CpRu-sulfoxide complexes requires a six-step sequence that was developed in our group [44]. Scheme 3 outlines the synthetic sequence of *p*-anisyl catalyst **1**. In situ reduction of 4-methoxysulfonyl chloride by triphenylphosphine and trapping with (−)-menthol affords diastereomerically pure sulfinate ester **2** after enrichment by recrystallization [45]. Grignard addition attaches a TMS-protected alkyne of appropriate tether length via stereospecific nucleophilic displacement of the chiral auxiliary with complete inversion of configuration at the sulfur center [46–47]. Sulfoxide **3** is incorporated into the catalyst via a [3 + 2] oxidative cycloaddition with allylruthenium complex **4**. Desilylation of cationic complex **5**, ion exchange, and photolysis of **6** completes the synthesis of catalyst **1**. Using this strategy, a variety of catalysts including *p*-tolyl, 2-naphthyl, 1-naphthyl, and *tert*-butyl sulfoxide complexes were all synthesized in an analogous fashion. This method has a distinct advantage over a traditional CpRu catalyst synthesis in that the complexes can be made quickly and efficiently without relying on toxic thallium salts to transfer substitutionally complex cyclopentadienyl ligands to ruthenium.

**Figure 2:**
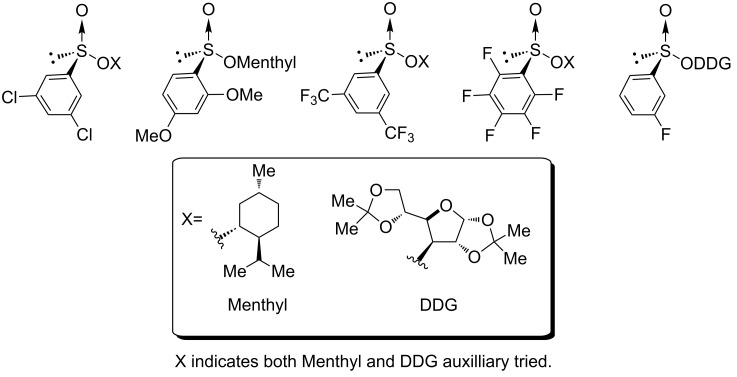
Failed sulfinate ester syntheses.

While certainly attractive, the main limitation to this synthetic route is that the diastereomeric mixture of sulfinate esters made in the first step is required to either be a solid that can be recrystallized to diastereomeric purity or be separable by column chromatography. Figure 2 contains the attempted sulfinate ester syntheses that failed using the method described in Scheme 3. An alternate method to synthesize these chiral sulfoxides needed to be explored, preferably one that did not rely on crystallization.

**Scheme 4:**
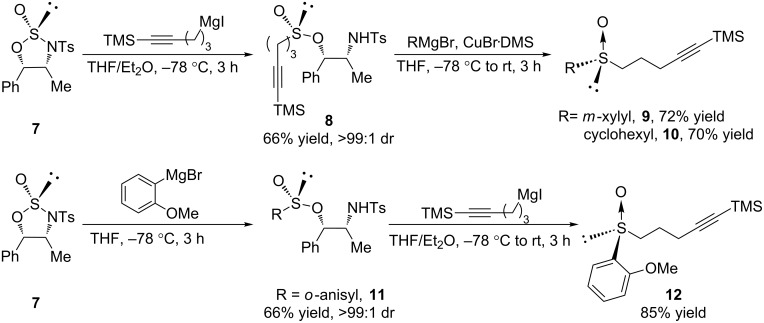
Using norephedrine-based oxathiazolidine-2-oxide **7** for chiral sulfoxide synthesis.

In 2005, the Senanayake group described a process in which (+)-norephedrine-derived oxathiazolidine 2-oxides are used as sulfinyl transfer agents in the synthesis of optically pure sulfoxides and sulfinamides [48]. The application of this method to the synthesis of our chiral sulfoxide tethers is presented in Scheme 4. Tosyl protection of the primary amine of (+)-norephedrine and treatment with thionyl chloride furnishes chiral oxathiazolidine 2-oxide **7** as a single diastereomer in 87% yield over two steps. Heterocycle **7** is a bench stable white powder that can be stored indefinitely in a dessicator without any noticeable decomposition. The sulfonamide moiety activates sulfur towards nucleophilic addition, making the first addition of an organometallic reagent faster than the second. By performing two successive organometallic additions to **7**, one could in principle obtain any desired chiral sulfoxide. Addition of a slight excess of (5-trimethylsilyl)-4-pentynylmagnesium iodide to the auxiliary at −78 °C affords sulfinate ester **8** in a 66% yield and as a single diastereomer. Organocuprate addition to **8** completes the synthesis of tether precursors **9** and **10**. The use of an organocuprate is essential in order to obtain good yields of the desired sulfoxides; the enantiospecificity of this organocuprate addition was checked by comparison of the optical rotation of **3** synthesized by this method with **3** synthesized by the menthyl sulfinate ester method used in Scheme 3. The o-anisyl sulfoxide **12** had to be synthesized in a slightly different manner because the organocuprate addition to **8** failed, most likely due to deactivation of the organometallic by the proximal methoxy group. Instead, sulfinate ester **11** was synthesized and subjected to alkylation with (5-trimethylsilyl)-4-pentynylmagnesium iodide. Because the order of addition was reversed, it is important to note that **12** has the opposite absolute configuration. In this way, sulfoxides containing *m-*xylyl, *o*-methoxyphenyl, and cyclohexyl groups have been made and carried through the remainder of the standard catalyst synthesis as outlined in Scheme 3. None of the catalysts made through this method could be synthesized through separation of sulfinate ester diastereomers.

### Substrate synthesis

**Scheme 5:**
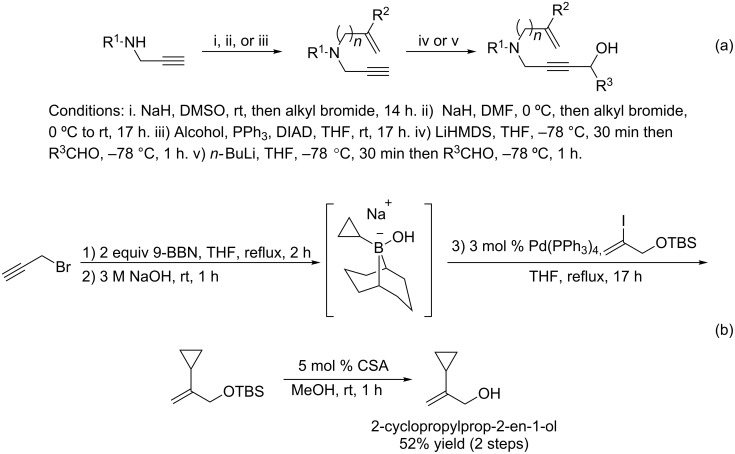
(a) General synthetic sequence to access enyne bicycloisomerization substrates (b) Synthesis of 2-cyclopropylprop-2-en-1-ol using a modified Suzuki coupling reaction developed by Soderquist.

The substrates for the enyne bicycloisomerization reaction can be readily accessed in two steps. Alkylation of a secondary propargylamide can be achieved by sodium hydride deprotonation of its acidic proton and S_N_2 substitution of a substituted propargyl bromide (Scheme 5a). Alternatively, the same propargylamide can be alkylated under Mitsunobu conditions with a desired primary alcohol. One such alcohol, 2-cyclopropylprop-2-en-1-ol, can be synthesized using a modified Suzuki coupling procedure developed by Soderquist [49] (Scheme 5b). A cyclopropyl boronate can be generated from propargyl bromide, 9-BBN, and aqueous sodium hydroxide. This reactive intermediate can be used in situ for the subsequent coupling reaction, which constructs the desired allyl alcohol after deprotection in a 52% yield over two steps. After alkylation, the newly-formed enynes can then be deprotonated with either *n-*BuLi or LiHMDS and trapped with an aldehyde to form a substituted propargyl alcohol. With a convenient route towards these enyne substrates in hand, we set our sights on optimizing the asymmetric enyne bicycloisomerization reaction.

### Initial experiments and reaction optimization

Due to the similarity of **14** to the triple-uptake inhibitor GSK1360707 (see Scheme 2), we decided to initiate our efforts on 1,7-enyne sulfonamide **13** for reaction optimization. Table 1 showcases our initial experiments. With 3 mol % of CpRu-sulfoxide catalyst **1** in THF at 40 °C, **14** could be obtained in a 69% yield and a promising 26.5:73.5 er (Table 1, entry 1). This important first experiment establishes that **1** not only efficiently catalyzes redox bicycloisomerization, but also that the ligated chiral sulfoxide can induce asymmetry in the [4.1.0] bicyclic product. Indeed, we have shown through control experiments that CpRu(MeCN)_3_PF_6_ does not catalyze this reaction without added ligands, implying that the sulfoxide must be bound to the metal in order for the reaction to proceed. Raising the temperature to 60 °C had a negligible impact on enantioselectivity (Table 1, entry 2).

**Table 1:** Initial result for the asymmetric redox bicycloisomerization of 1,7-enyne **13** with chiral CpRu-sulfoxide complex **1** and the effect of added ligands.

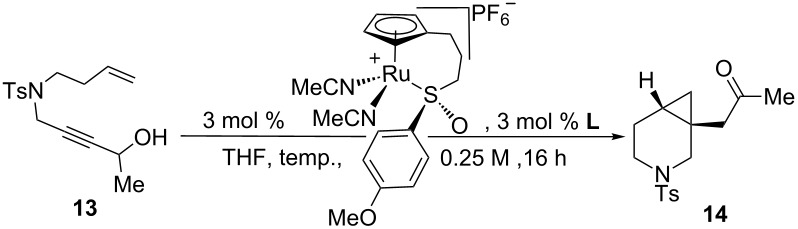					

entry	ligand	temp. (°C)	conversion (%)^a^	yield (%)	er^b^

1	none	40	100	69	26.5:73.5
2	none	60	100	89	26.0:74.0
3	PPh_3_	60	0	n.r.	–
4	P(OPh)_3_	60	0	n.r.	–
5	(+)-(*R*)-methyl *p*-tolyl sulfoxide	60	100	74	28.0:72.0
6	(+)-(*R*)-methyl *p*-tolyl sulfoxide	40	50	22	41.5:58.5
7	(−)-(*S*)-methyl *p*-tolyl sulfoxide	40	0	N.R.	–
8	(±)-*N*-(*p*-tosyl) methyl *p*-tolyl sulfimide	40	50	22	50.0:50.0

^a^Determined by NMR integration. ^b^Determined by chiral HPLC.

Our proposed mechanism of the racemic redox bicycloisomerization reaction necessitates the decomplexation of one phosphine ligand before catalysis can occur (vide supra). In other words, ruthenium must have two open coordination sites in order to bind the substrate. To test if there is a similar requirement for **1**, we decided to add one catalyst equivalent of an auxiliary ligand to test its impact on reaction rate, conversion, and enantioselectivity. Added phosphorous-based ligands only served to completely shut down all reactivity (Table 1, entries 3 and 4). At 60 °C, 3 mol % of chiral (+)-(*R*)-methyl *p*-tolyl sulfoxide had no impact on the reaction, indicating negligible binding of the ligand to the metal (Table 1, entry 5). In general, untethered, exogenous sulfoxides are poorer ligands to ruthenium than phosphines or phosphites. As the reaction temperature is lowered to 40 °C, however, one begins to see a significant decrease in reaction rate and conversion (Table 1, entry 6). The enantioselectivity of the reaction also drops to 41.5:58.5 er Interestingly, (−)-(*S*)-methyl *p*-tolyl sulfoxide completely inhibited any reaction at 40 °C (Table 1, entry 7). Finally, the more electron-withdrawing (±)-*N*-*p*-tosyl methyl *p*-tolyl sulfimide (*p*-CH_3_Ph(S=NTs)CH_3_) also reduced both conversion and er.

As seen in Table 2, the redox bicycloisomerization reaction is compatible with a number of solvents that have vastly different steric profiles and dielectric constants. The nature of the solvent also had a significant impact on the enantiomeric ratios. Switching from THF to acetone, we observed a dramatic reversal in enantioselectivity, going from 26.5:73.5 to 67.0:33.0, respectively (Table 2, entry 2). Changing the reaction temperature had no impact on the er, similar to the temperature studies done in THF (Table 2, entries 3 and 4).

**Table 2:** Solvent effects on asymmetric redox bicycloisomerization reaction as described in [43].

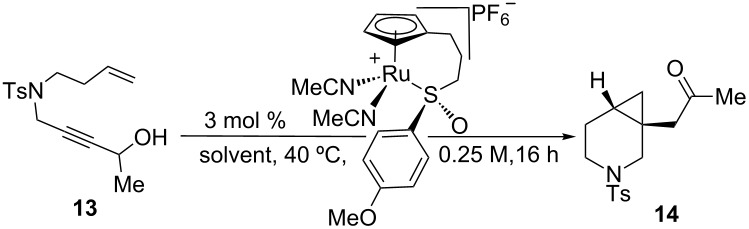				

entry	solvent	conversion (%)^a^	yield (%)	er^b^

1	THF	100	69	26.5:**73.5**
2	acetone	100	72	**67.0**:33.0
3^c^	acetone	100	69	**67.0**:33.0
4^d^	acetone	91	n.d.^e^	**66.0**:34.0
5^c^	2,5-Me_2_THF^f^	50	17	26.5:**73.5**
6^c^	iPr Me ketone	70	47	**57.5**:42.5
7	DCE	100	66	**60.5**:39.5
8	toluene	59	n.d.^e^	31.5:**68.5**
9	EtOAc	100	65	37.0:**63.0**
10	MeOH	100	80	45.0:**55.0**
11	1,4-dioxane	100	59	32.0:**68.0**
12	DME	100	56	27.0:**73.0**
13	THF^g^	62	13	41.0:**59.0**
14	acetone^g^	88	56	**61.0**:39.0

^a^Determined by NMR integration. ^b^Determined by chiral HPLC. ^c^Reaction performed at 60 degrees. ^d^Reaction performed at room temperature. ^e^Not determined due to inseparability from starting substrate. ^f^Mixture of *cis* and *trans*. ^g^2 vol % DMF added.

Initially, we thought that this difference in selectivity between acetone and THF was due to their drastically different steric profiles, so we tested bulkier analogues of these solvents, 2,5-dimethyl THF (mixture of *cis* and *trans*, entry 5) and 3-methyl-2-butanone (Table 2, entry 6). Complex **1** catalyzed the redox bicycloisomerization reaction less efficiently in both solvents. Chlorinated (Table 2, entry 7), aromatic (entry 8), ester (entry 9), and alcohol (entry 10) solvents were all tried, with inferior results to acetone and THF and without any obvious trends in selectivity apparent. Finally, alternative ethereal solvents like 1,4-dioxane (Table 2, entry 11) and DME (entry 12) were checked, as well as the effect of adding a small volume percentage of *N*,*N*-dimethylformamide (Table 2, entries 13 and 14). No improvement in enantioselectivity was observed.

The effect of catalyst structure on the selectivity of the redox bicycloisomerization reaction was explored in both acetone and THF, due to the differential nature of both of these solvents (Table 3). *p*-Tolyl and 2-naphthyl sulfoxide complexes **15** and **16** were both able to catalyze the reaction to complete conversion, although the enantiomeric ratios of **14** were lower than in the *p*-anisyl case. Curiously, the bulkier 1-naphthyl sulfoxide complex **17** was completely ineffective, most likely due to deligation of the sulfoxide prior to coordination of the substrate (Table 3, entry 4). The more electron-rich, bulky *t*-butyl sulfoxide catalyst **18**, was able to maintain coordination to ruthenium, though the enantiomeric ratios obtained were unsatisfactory (Table 3, entry 5). *m*-Xylyl **19** was similarly disappointing (Table 3, entry 6). However, *o*-anisyl **20** was interesting in that it was able to maintain high enantiomeric ratios of **14** in both THF and acetone and with the same absolute configuration (Table 3, entry 7). This could possibly be due to a chelation effect wherein the *o*-methoxy group acts as a hemilabile ligand, changing the steric and electronic environment around the metal center. Since **20** displayed inferior conversions and yields, however, it was eschewed in favor of complex **1**. Cyclohexyl **21** was also an excellent catalyst in terms of reactivity, but again this system did not prove to be differential towards the enantiomeric ratio of **14**.

**Table 3:** Effect of the catalyst structure on the redox bicycloisomerization as described in [43].

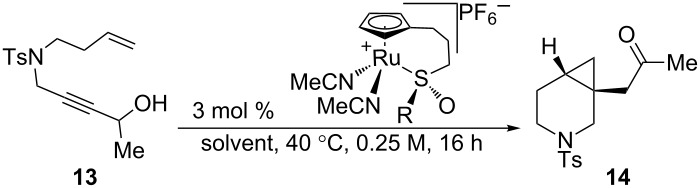					

entry	R	solvent	conversion (%)^a^	yield (%)	er^b^

1	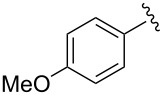 **1**	acetone THF	100 100	72 69	**67.0**:33.0 26.5:**73.5**
2	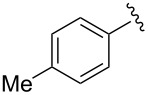 **15**	acetone THF	100 100	72 53	**64.5**:35.5 41.0:**59.0**
3	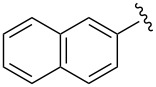 **16**	acetone THF	100 100	72 58	**60.5**:39.5 31.5:**68.5**
4	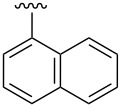 **17**	acetone THF	0 0	n.r. n.r.	– –
5	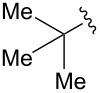 **18**	acetone THF	100 93	69 44	40.5:**59.5** 43.5:**56.5**
6	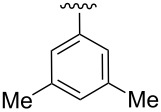 **19**	acetone THF	50 82	11 36	**52.0**:48.0 **54.0**:46.0
7^c^	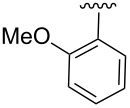 **20**	acetone THF	100 63	50 30	27.5:**72.5** 22.0:**78.0**
8	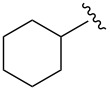 **21**	acetone THF	100 100	70 57	**73.5**:26.5 47.5:**52.5**

^a^Determined by NMR integration. ^b^Determined by chiral HPLC. ^c^Catalyst enantiomer opposite to the one shown.

Before moving forward with complex **1**, we wondered how these complexes compare to other catalyst/ligand systems in terms of reactivity (Table 4). After all, complexing an achiral, commercially available catalyst CpRu(MeCN)_3_PF_6_ with a chiral ligand would constitute a much simpler solution to the development of an asymmetric redox bicycloisomerization reaction. To compare the efficiency of each catalyst system, we decided to run the reaction for a shorter period of time, stop the reaction, and check the conversion by proton NMR.

**Table 4:** Comparison of complex **1** to other catalyst systems as described in [43].

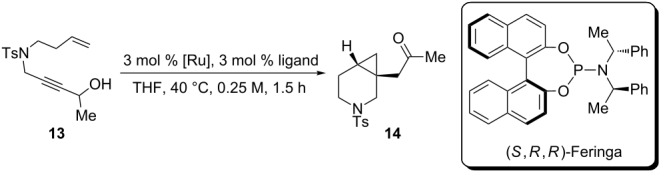			

entry	[Ru]	ligand	conversion (%)^a^

1	**1**	none	54
2	CpRu(MeCN)_3_PF_6_	PPh_3_	15
3	CpRu(MeCN)_3_PF_6_	(*R*)-BINAP	0
4	CpRu(MeCN)_3_PF_6_	(*S*,*S*,*R*)-Feringa	0
5	CpRu(MeCN)_3_PF_6_	(*S*)-methyl *p*-tolyl sulfoxide	0

^a^Determined by ^1^H NMR.

As seen in Table 4, when this experiment is performed with 3 mol % complex **1**, a 54% conversion is observed after 1.5 hours (entry 1). When the same experiment is conducted with 3 mol % of CpRu(MeCN)_3_PF_6_ and 3 mol % triphenylphosphine, the reaction only proceeds to 15% conversion, indicating that the tethered sulfoxide complex is much more efficient at catalyzing redox bicycloisomerization (Table 4, entry 2). As one would expect from the lessons learned in the previous studies, chiral bidentate (*R*)-BINAP completely inhibits any reactivity (Table 4, entry 3). Surprisingly, monodentate (*S,S,R*)-Feringa was also an ineffective ligand for this process, which underscores the stringent electronic requirements a ligand must have in order to promote redox bicycloisomerization (Table 4, entry 4). To date, the only effective ligands for redox bicycloisomerization have been triaryl phosphines, biaryl sulfoxides, or monoaryl monoalkyl sulfoxides. After 1.5 hours, (*S*)-methyl *p*-tolyl sulfoxide also showed no conversion (Table 4, entry 5).

Since subtle changes in the structure of the enyne substrate could have a significant impact on enantioselectivity, variations of the substituent on nitrogen were examined (Table 5). These reactions were conducted in THF, as this solvent provided the highest enantioselectivities in the [4.1.0] system. Incorporating electron rich or electron poor aromatics on the sulfonamide resulted in diminished reactivities and selectivities, as seen in the cases of entries 2 and 3 (Table 5). Surprisingly, 2-biphenyl sulfonamide **26** gave an unimproved 26.0:74.0 er, despite the increased steric bulk on the substrate (Table 5, entry 4). Further increasing the Lewis basicity of the nitrogen completely shuts down catalyst activity, as can be seen in the case of the benzhydryl tertiary amine **28** (Table 5, entry 5). Phosphoramidate **31** could be obtained in good yield and with an increased enantioselectivity when compared to the parent sulfonamide (Table 5, entry 6). Amide **32** also reacted, but with a lower conversion and yield, possibly due to increased coordination of the amide to the Lewis acidic ruthenium center (Table 5, entry 7). Gratifyingly, utilizing the bulkier 2,4,6-triisopropylbenzenesulfonyl group (Tris) significantly improved the er of protected piperidine **35** to 15.0:85.0. Because of its differential impact on selectivity, this protecting group was pursued more broadly in the substrate scope.

**Table 5:** Effect of the nitrogen protecting group on reactivity and enantioselectivity as described in [43].

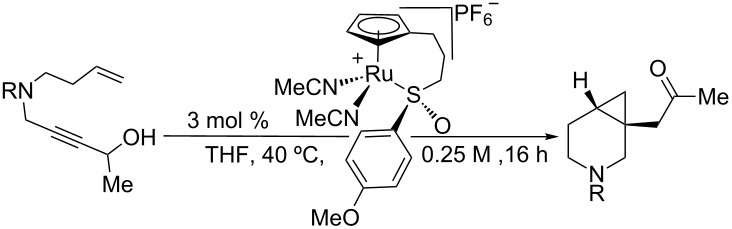					

entry	R	substrate	product	yield (%)	er^a,b^

1	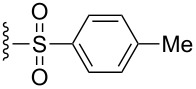	**13**	**14**	69	26.5:**73.5**
2	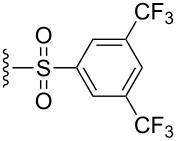	**22**	**23**	21	31.5:**68.5**
3	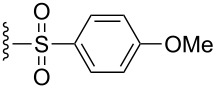	**24**	**25**	n.d.^c^	43.5:**56.5**
4	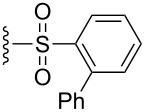	**26**	**27**	59	26.0:**74.0**
5	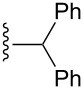	**28**	**29**	n.r.^d^	–
6	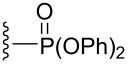	**30**	**31**	52	21.0:**79.0**
7	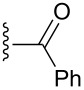	**32**	**33**	45^e^	26.5:**73.5**
8	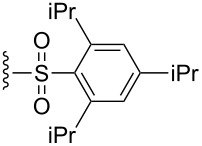	**34**	**35**	56	15.0:**85.0**

^a^Determined by chiral HPLC. ^b^(Enantiomer A:Enantiomer B). Absolute configuration not determined. Bold indicates major enantiomer. ^c^n.d.= not determined. Conversion ~30%. ^d^n.r. = no reaction. ^e^Conversion ~50%.

### Substrate scope

Unfortunately, the conditions developed for the Tris-protected [4.1.0] bicycle **35** did not extend to other similarly protected 1,7-enynes [43]. We decided to shift our focus from 1,7- to 1,6-enynes to determine if bicyclic [3.1.0] pyrrolidines are more broadly accessible to our synthetic method (Table 6). While the 1,6-enyne substrates **36** and **38** exhibited the desired reactivity in THF, the enantioselectivities for the process were poor. Considering how impactful a judicious choice of solvent had on the enantioselectivity of the [4.1.0] bicycles, we decided to try the same reactions in acetone. Pleasingly, exceptional yields and enantioselectivities of **37** and **39** were obtained in this solvent, surpassing those obtained for **35**. Catalyst **1** exhibits excellent functional group compatibility. Substrates containing remote electron-neutral aromatic rings **40**, alkenes **42**, and alkynes **44** are all tolerated and remain intact under the reaction conditions. The reaction can also tolerate branching at the internal position of the pendant olefin, as polycyclic **46** and **48** displayed excellent enantiomeric ratios. It is also important to point out that no ring opening of either **49** or its vinylcyclopropane precursor **48** was observed. Finally, styrenyl substrates like **50** can be used for redox bicycloisomerization, but their pyrrolidine products are isolated in somewhat diminished enantioselectivities.

**Table 6:** Tris-protected [3.1.0] bicyclic pyrrolidines made by redox bicycloisomerization^a^.

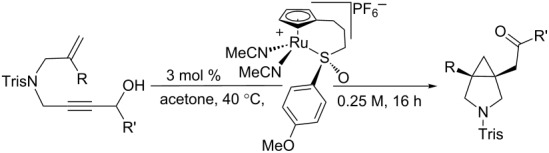						

entry	R	R’	enyne	product	Yield (%)	er

1	Me	Me	**36**	**37**	78 85	56.0:44.0^b^ 90.5:9.5
2	Me	iPr	**38**	**39**	47 75	64.0:36.0^b^ 98.5:1.5
3^c^	Me	Bn	**40**	**41**	88	92.0:8.0
4	Me	-(CH_2_)_8_CH=CH_2_	**42**	**43**	57	93.0:7.0
5^d^	Me	-(CH_2_)_4_C≡CH_3_	**44**	**45**	48	90.0:10.0
6	cyclopentyl	iPr	**46**	**47**	42	94.0:6.0
7	cyclopropyl	cyclohexyl	**48**	**49**	75	90.0:10.0
8	*p*-tolyl	Me	**50**	**51**	50	82.5:17.5

^a^Tris = 2,4,6-triisopropylbenzenesulfonyl. ^b^Reaction conducted in THF. ^c^5 mol % of catalyst used. ^d^8.5 mol % of catalyst used.

After examining the scope of the redox bicycloisomerization reaction for the synthesis of Tris-protected [3.1.0] pyrrolidines, it occurred to us that protecting groups other than Tris may be equally effective for the [3.1.0] system. The Tris group was chosen during our optimization of the six-membered ring system (Table 5), which may have significantly different requirements necessary to achieve high enantioselectivity. Indeed, when tosyl- or phosphoramidate-protected 1,6-enynes were tested, excellent yields and enantioselectivities were observed (Table 7). Like the Tris substrates, branching at the propargylic and alkenyl positions were well tolerated. Carbocyclic **69** could also be isolated with similar results, although the catalyst loading for this substrate had to be increased to 7.5 mol %. This is most likely due to the tendency of ruthenium to coordinate to certain aromatic rings in a η^6^ fashion.

**Table 7:** Other substrate tethers for redox bicycloisomerization.

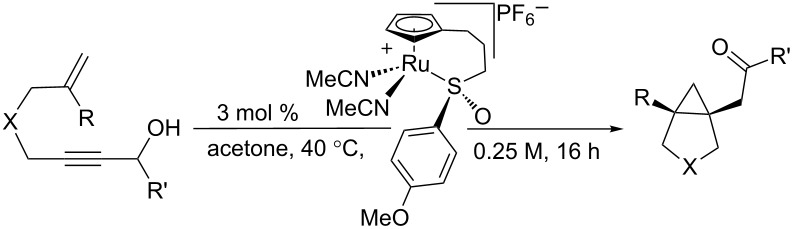							

entry	X	R	R’	enyne	product	yield (%)	er

1	-NTs	Me	Me	**52**	**53**	81	94.0:6.0
2	-NTs	Me	Bn	**54**	**55**	84	95.0:5.0
3	-NTs	cyclopentyl	iPr	**56**	**57**	56	91.0:9.0
4	-NP(O)(OPh)_2_	Me	Me	**58**	**59**	84	98.0:2.0
5^a^	-NP(O)(OPh)_2_	Me	Bn	**60**	**61**	61	97.0:3.0
6	-NP(O)(OPh)_2_	cyclopentyl	iPr	**62**	**63**	64	96.0:4.0
7	-NP(O)(OPh)_2_	Me	cyclohexyl	**64**	**65**	51	96.0:4.0
8	-NP(O)(OPh)_2_	Ph	Me	**66**	**67**	69	88.0:12.0
9^b^	-C(CO_2_Bn)_2_	Me	iPr	**68**	**69**	49	92.0:8.0

^a^5 mol % of catalyst used. ^b^7.5 mol % of catalyst used.

**Figure 3:**
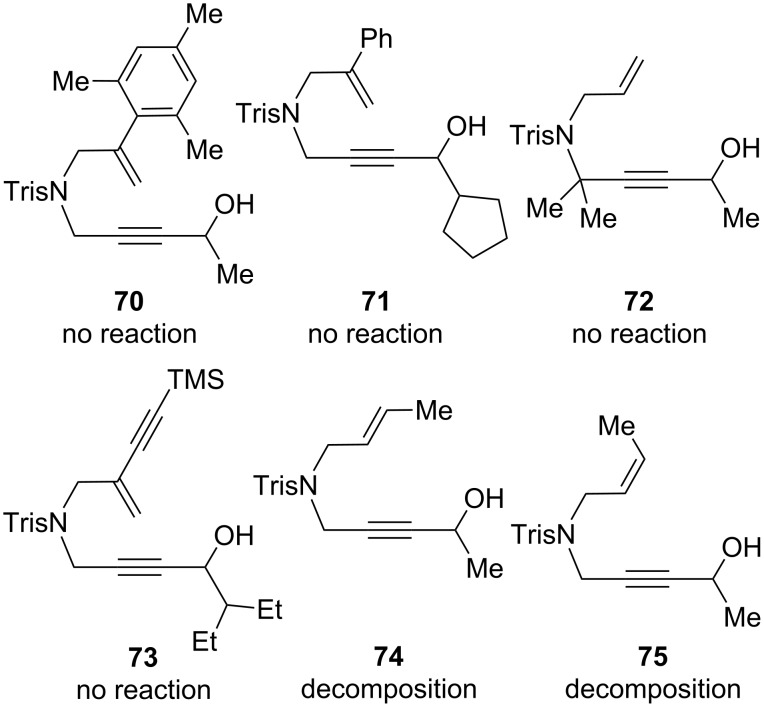
Failed bicycloisomerization substrates. Reactions performed at 40 °C for 16 hours with 3 mol % of catalyst **1** in acetone at a 0.25 M concentration relative to substrate.

There were a few substrate classes that were not amenable to redox bicycloisomerization (Figure 3). For example, we thought that by increasing the steric bulk on styrenyl-substituted compound **50**, either on the aromatic ring or at the propargylic position, the enantioselectivity would also increase. On the contrary, neither substrate **70** or **71** reacted at all, which indicated to us that this substrate class is very sensitive to steric substitution and where it is placed on the enyne. In other words, while both phenyl substitution and branching at the propargylic position are tolerated on separate enyne substrates (see for example **48** and **50**), putting both moieties on the same substrate results in no reaction. Increasing steric congestion near the sulfonamide (substrate **72**) also inhibits enyne bicycloisomerization, as does placing an additional alkyne in conjugation with the pendant alkene (substrate **73**). Both *cis-* and *trans*-1,2-disubstituted alkenes **74** and **75** were observed to decompose under the standard reaction conditions.

**Scheme 6:**
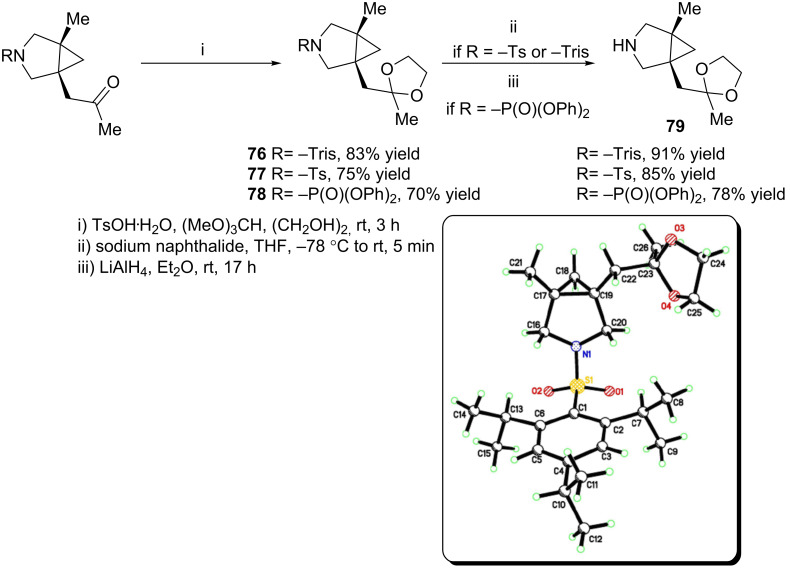
Deprotection of [3.1.0] bicycles and X-ray crystal structure of **76**.

The tosyl and 2,4,6-triisopropylbenzenesulfonyl groups on nitrogen can be removed after protection of the ketone as the cyclic acetal by using sodium naphthalide in THF (Scheme 6). After protection, the diphenylphosphoramidate group can also be removed with lithium aluminum hydride in excellent yield. The absolute configuration of the [3.1.0] pyrrolidines was assigned by analogy to **76**, which was determined to be (*R*,*R*) by single crystal X-ray crystallography.

### Mechanistic studies

**Scheme 7:**
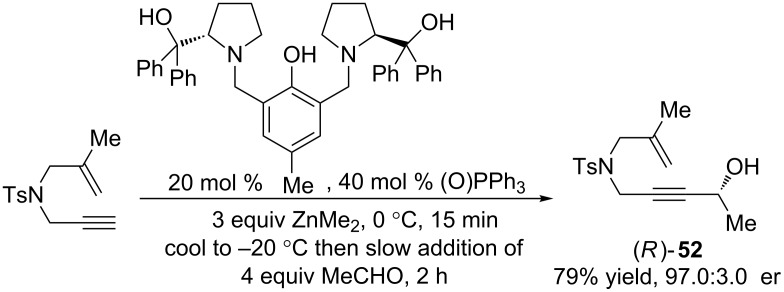
ProPhenol-catalyzed addition of zinc acetylide to acetaldehyde for the synthesis of a chiral 1,6-enyne substrate.

After having successfully developed this synthetic methodology, a few questions still remained pertaining to the mechanism of the reaction. First, does the propargylic stereocenter on the enyne substrate have a significant impact on the enantioselectivity of the reaction, despite it being destroyed during redox isomerization? Second, what role does the nature of the solvent play in determining enantioselectivity? To answer these questions, we synthesized a 1,6-enyne containing an enantioenriched propargyl alcohol using our group’s zinc ProPhenol chemistry (Scheme 7). By employing the opposite enantiomers of the ProPhenol catalyst, either enantiomer of propargyl alcohol can be accessed with this methodology. Additionally, no other attempted synthetic strategy was able to provide (*R*)-**52**, including Noyori and CBS reduction, which highlights the utility of this asymmetric transformation.

In acetone, the nature of this stereocenter had very little impact on the enantiomeric ratio of the final product, as both (*R*)- and (*S*)-**52** gave results that were almost identical to the racemic substrate (Table 8). Interestingly, however, switching to THF provided drastically different results. While redox bicycloisomerization of (*R*)-**52** was very enantioselective, affording **53** in a 92.0 to 8.0 enantiomeric ratio, (*S*)-**52** performed much worse. Product **53** was isolated in a 68.0 to 32.0 enantiomeric ratio in this case. The same matched/mismatched effect was observed for chiral 1,7-enynes in THF [44]. Based on these results, it is clear that the high enantioselectivity of the redox bicycloisomerization reaction of 1,6-enynes is due to acetone’s ability to override any impact the propargylic stereocenter has on the course of the reaction.

**Table 8:** Effect of propargylic stereocenter on enantioselectivity.

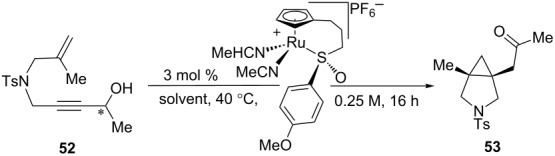				

entry	solvent	alcohol configuration	yield (%)	er

1	acetone	*rac*	81	94.0:6.0
2	acetone	(*R*)^a^	84	93.5:6.5
3	acetone	(*S*)^a^	81	96.5:3.5
4	THF	*rac*	52	80.0:20.0
5	THF	(*R*)^a^	61	92.0:8.0
6	THF	(*S*)^a^	52	68.0:32.0

^a^Starting alcohol has a 97.0:3.0 er.

**Figure 4:**
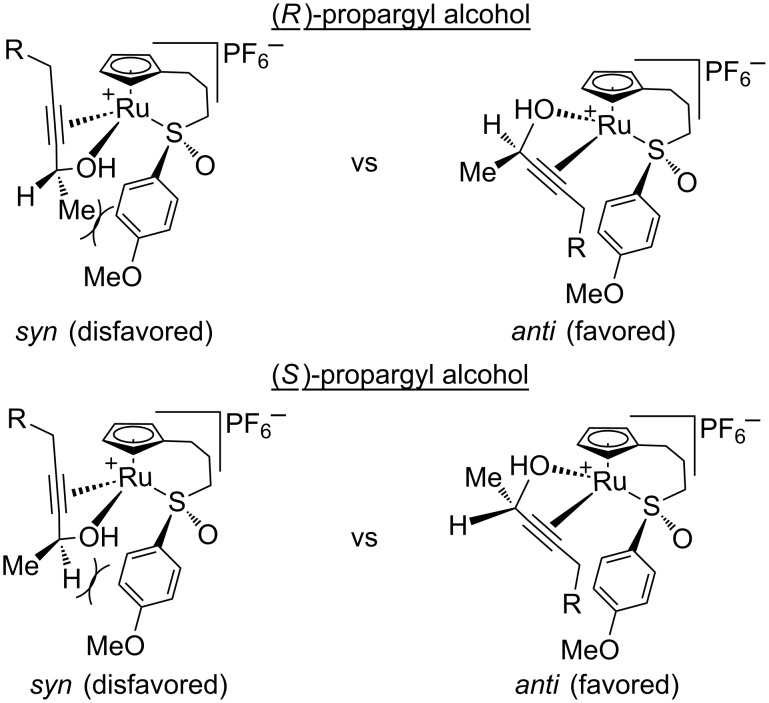
Diastereomeric metal complexes formed after alcohol coordination.

Considering the data presented in Table 8, we now propose a working mechanism for the origin of enantioselectivity in the redox bicycloisomerization reaction. There are two important factors to consider when putting together such a mechanism: coordination of the propargyl alcohol on the metal center and facial selectivity for the [2 + 2] cycloaddition. We will look at each in turn. First, coordination by the propargyl alcohol creates four possible diastereomeric, chiral-at-ruthenium intermediates (Figure 4). As long as the metal does not epimerize over the course of the reaction, this transfer of stereochemical information can then be transferred back to the substrate following redox isomerization, influencing the overall enantioselectivity of the process. For both the (*R*)- and the (*S*)-propargyl alcohols, the CpRu-sulfoxide catalyst prefers to place the stereocenter away from the steric bulk of the sulfoxide (*anti* configuration). Both *syn* configurations are disfavored, but the *syn-*(*R*)-configuration is energetically more taxing than the *syn-*(*S*)-configuration, creating a larger energy difference between *syn-*(*R*) and *anti-*(*R*). This larger energy difference is reflected in the higher enantioselectivities obtained for the (*R*) enantiomer in THF (Table 8, entry 5). The smaller energetic difference between *syn-*(*S*) and *anti*-(*S*) means that there is less of a preference for either coordination mode, which leads to poorer enantioselectivities in THF (Table 8, entry 6).

**Scheme 8:**
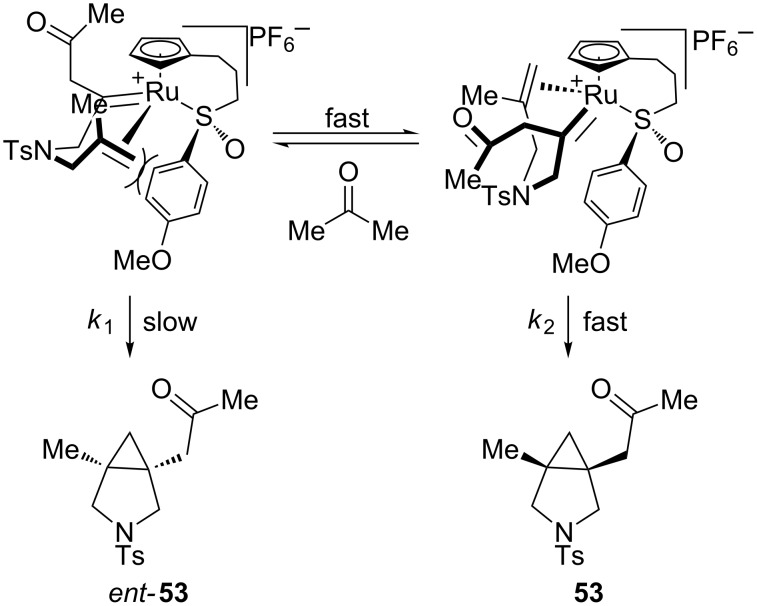
Curtin–Hammitt scenario of redox bicycloisomerization in acetone.

We propose that acetone, being more Lewis basic than THF, has the effect of epimerizing the chiral-at-ruthenium intermediates formed prior to [2 + 2] cycloaddition. The rate of epimerization is much faster than the [2 + 2] cycloaddition, creating a classic Curtin–Hammitt scenario wherein all of the substrate is funneled into the observed enantiomer of product (Scheme 8). Rate *k*_1_ is much slower than *k*_2_ due to the severe steric hindrance imposed by the ligated chiral sulfoxide, which block alkene coordination. The pendant olefin prefers to approach the carbene *anti* to the aforementioned sulfoxide, resulting in the observed enantiomer of **53**. In THF, the rate of epimerization is significantly slower than the [2 + 2] cycloaddition, which means that the enantiomeric ratios observed in the products are affected more by the initial coordination of the propargyl alcohol.

## Conclusion

In conclusion, we have developed a ruthenium-catalyzed asymmetric redox bicycloisomerization utilizing a chiral CpRu–sulfoxide complex **1**. This constitutes the first example of an asymmetric ruthenium-catalyzed redox isomerization known to date. We were able to demonstrate that the nitrogen protecting group on the 1,7-enynes had a significant impact on the enantioselectivity of the redox bicycloisomerization as did the choice of solvent. Extending the chemistry to the 1,6-enynes, we showed that these substrates were much more amenable to a wider range of groups on nitrogen, though a significant solvent effect was still observed in these cases. The mechanism of the reaction was then probed by performing the redox isomerization reaction on enantioenriched propargyl alcohols. While a significant matched/mismatched effect was observed in THF, no such effect was seen in acetone. We postulate that there are two different enantiodetermining steps that center around a chosen solvent’s ability to epimerize a metal center following redox isomerization.

## Supporting Information

File 1 Experimental details and NMR data of new catalysts.

File 2 X-ray crystallography data for **76**.
