# Supplementary material for: Chiral cyclopentadienylruthenium sulfoxide catalysts for asymmetric redox bicycloisomerization
Source: Beilstein J Org Chem. 2016 Jun 7;12:1136–52. doi: 10.3762/bjoc.12.110 (PMC4979649; doi:10.3762/bjoc.12.110)
Supplement: File 1 — Experimental details and NMR data of new catalysts. [file Beilstein_J_Org_Chem-12-1136-s001.pdf]

Supporting Information

for

Chiral cyclopentadienylruthenium sulfoxide catalysts for asymmetric redox bicycloisomerization

Barry M. Trost*[1], Michael C. Ryan[1], and Meera Rao[1]

Address: [1]Department of Chemistry, Stanford University, Stanford, California 94305-5580, USA

Email: Barry M. Trost - bmtrost@stanford.edu

*Corresponding author

This article dedicated to the memory of Prof. Peter Hofmann

Experimental details and NMR data of new catalysts

All reactions were performed in flame- or oven-dried glassware with magnetic stirring under a nitrogen or argon atmosphere using freshly distilled or column-dried solvents. All other commercial reagents were used without purification unless otherwise noted. Air and moisture sensitive liquids and solutions were transferred via stainless steel syringe or cannula and introduced into the reaction vessel through rubber septa. Reactions conducted below room temperature were cooled by an external bath: dry ice in acetone for –78 °C or ice in water for 0 °C. Thin-layer chromatography was performed on EMD silica gel 60 F254 plates (0.25 mm); visualization of the developed chromatogram was performed by fluorescence quenching and staining with aqueous ceric ammonium molybdate, *p*-anisaldehyde, or potassium permanganate. Organic solutions were concentrated by rotary evaporation below 40 °C at ca. 25 mm Hg. Chromatographic purification of products was accomplished using forced-flow chromatography on silica gel (particle size 0.040–0.063 mm). All isolated and characterized compounds were >90% pure as judged by 1H-NMR spectroscopic analysis unless noted otherwise. ^{1}H and ^{13}C NMR spectroscopy were performed on a 400 MHz NMR spectrometer Varian Mercury console Sun Blade 100 operating at 400 (^{1}H) and 100 MHz (^{13}C), respectively, as well as on a 300 MHz NMR

spectrometer Varian Inova console Sun Sparcstation 5 operating at 300 (^{1}H) and 75 MHz (^{13}C), respectively. Chemical shifts are reported in ppm relative to residual protio solvent signals; all ^{13}C NMR spectra are proton decoupled. Data for ^{1}H are reported as chemical shift (δ ppm), multiplicity (s = singlet, d = doublet, t = triplet, q = quartet, quint = quintet, sex = sextet, sept = septet, oct = octet, m = multiplet, app. = apparent), coupling constant, integration); data for ^{13}C are reported in terms of chemical shift. Infrared spectroscopic data was recorded on sodium chloride plates as thin films on a Thermo Scientific Nicolet IR100 FT-IR spectrometer. Mass spectrometry data were collected by LC/ESI-MS on a Waters Acquity UPLC and Thermo Fisher Exactive mass spectrometer scanning *m/z* 100–1200. The LC conditions were 95% Solvent A (0.1% formic acid in water) held 1 minute then ramped to 98% Solvent B (0.1% formic acid in acetonitrile) in 2 minutes, and held for 3 minutes. The column was a 2.1 × 30mm Zorbax C18 3.5 um and the flow rate was 0.4mL/min. Chiral HPLC analysis was performed on a Thermo Separation Products Spectra Series P-100 using Chiralcel R and Chiralpak R columns. Experimental procedures and spectral data for enyne starting materials, bicycloisomerization products, and nitrogen deprotection products can be found in ref. [1].

I. Synthesis of new catalysts

(1*S*,2*R*)-2-(4-Methylphenylsulfonamido)-1-phenylpropyl-(*S*)-5-(trimethylsilyl)pent-4-yne-1-sulfinate (8)

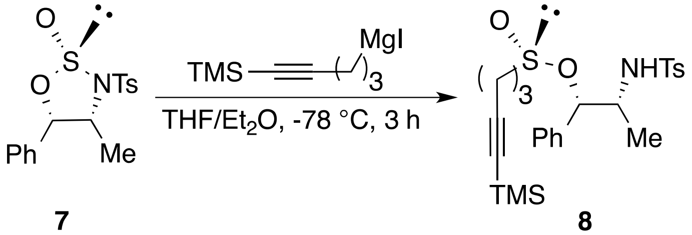

7 **8**

A 25 mL pear-shaped flask with stir bar was flame dried, charged with 1.12 g of (*S*)-N-tosyl-methyl-phenyl-1,2,3-oxathiazolidine-2-oxide **7** (3.19 mmol, 1 equiv), fitted with a septum, and placed under argon. 10.15 mL of THF from the solvent system was added via syringe to create a 0.31 M solution. The solution was cooled to −78 °C. 4.35 mL of a 0.77 M solution of (5-trimethylsilyl)-4-pentynlmagnesium iodide in Et_2O was added dropwise via syringe to the oxathiazolidine-2-oxide solution. The reaction was allowed to stir at −78 °C for 3 hours. After 3 hours, the reaction was quenched at −78 °C with sat'd aq. $NaHCO_3$ (5 mL). The reaction was diluted with 5 mL of EtOAc and allowed to warm to room

temperature. The phases were separated and the aqueous phase was washed two times with EtOAc (10 mL ea.). The combined organics were dried over Na_2SO_4, filtered, and concentrated in vacuo. The crude reaction mixture was purified via silica gel chromatography (3:1 hexanes/EtOAc) to give **8** as a colorless oil (1.03, 2.1 mmol, 66% yield). [R_f = 0.20 (3:1 hexanes/EtOAc)]; $[\alpha]_D^{25}$= 17.86° (1.0, $CHCl_3$); IR (thin film) ν_{max}= 2959, 2924, 2854, 2174, 1452, 1335, 1250, 1163, 1116, 1092, 843; ^{1}H NMR (400 MHz, $CDCl_3$) δ 7.84 (d, *J*= 8.4 Hz, 2H), 7.36-7.29 (m, 5H), 7.11 (d, *J*= 8.4 Hz, 2H), 5.53 (d, *J*= 9.6 Hz, 1H), 5.30 (s, 1H), 3.67 (m, 1H), 2.98 (m, 2H), 2.42 (s, 3H), 2.38 (m, 2H), 1.95 (m, 2H), 0.97 (d, *J*= 7.2 Hz, 3H); ^{13}C NMR ($CDCl_3$) δ 143.35, 138.31, 137.14, 129.72, 128.58, 128.31, 127.15, 125.69, 104.66, 86.61, 85.12, 55.96, 54.27, 21.54, 20.04, 19.03, 14.69, 0.01; LR-MS (m/z): [M + Na^+] calcd for $C_{24}H_{33}NNaO_4S_2Si$: 514.2; found: 514.4.

(*R*)-(5-((3,5-Dimethylphenyl)sulfinyl)pent-1-yn-1-yl)trimethylsilane (9)

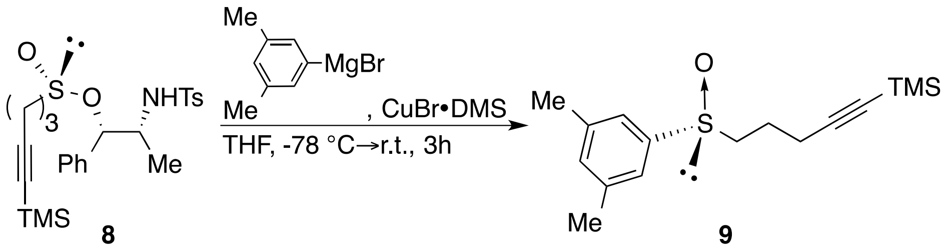

8 9

A 25 mL pear-shaped flask with stir bar was flame-dried and charged with copper (I) bromide-dimethyl sulfide complex (206 mg, 1 mmol, 5 equiv) and placed under argon. 1 mL of THF was added and the suspension was cooled to −78 °C. While the suspension sirred at −78 °C, 1.13 mL of a 1.62 M solution of 3,5-dimethylphenylmagnesium bromide in THF (1.8 mmol, 9 equiv) was added dropwise via syringe. The reaction mixture turned a bronze color while stirring at −78 °C for 30 minutes. After 30 minutes, 1 mL of a 0.2 M solution of **8** in THF (98 mg, 0.2 mmol, 1 equiv) was added dropwise via syringe. The reaction stirred for 1 hour at −78 °C and warmed to room temperature where it stirred for two hours. After two hours, the reaction was quenched with 2 mL of sat'd. aq. NH_4Cl and diluted with 2 mL of EtOAc. The layers were separated and the aqueous phase was washed two times with EtOAc (4 mL each). The combined organics were dried over $MgSO_4$, filtered, and concentrated in vacuo. Crude reaction mixture was purified via silica gel chromatography (10:1:0.5 hexanes/EtOAc/iPrOH). Sulfoxide **9** obtained as a pale yellow oil (42 mg, 0.14

mmol, 72% yield). [R_f = 0.17 (4:1:0.2 hexanes/EtOAc/MeOH)]; $[\alpha]_D^{25}$= 82.71° (1.0, $CHCl_3$); IR (thin film) ν_{max}= 2958, 2922, 2860, 2174, 1606, 1581, 1453, 1249, 1102, 1055, 909, 843, 760, 686, 638; ^{1}H NMR (400 MHz, $CDCl_3$) δ 7.21 (s, 2H), 7.10 (s, 1H), 3.00-2.91 (m, 1H), 2.90-2.82 (m, 1H), 2.45-2.31 (m, 8H), 2.02-1.92 (m, 1H), 1.88-1.77 (m, 1H), 0.13 (s, 9H); ^{13}C NMR ($CDCl_3$) δ 143.45, 139.21, 132.71, 121.39, 105.06, 86.27, 55.91, 21.29, 21.15, 18.87, 0.04; LR-MS (m/z): [M + Na^+] calcd for $C_{16}H_{24}NaOSSi$: 315.1; found: 315.3.

***m*-Xylyl sulfoxide catalyst (19)**

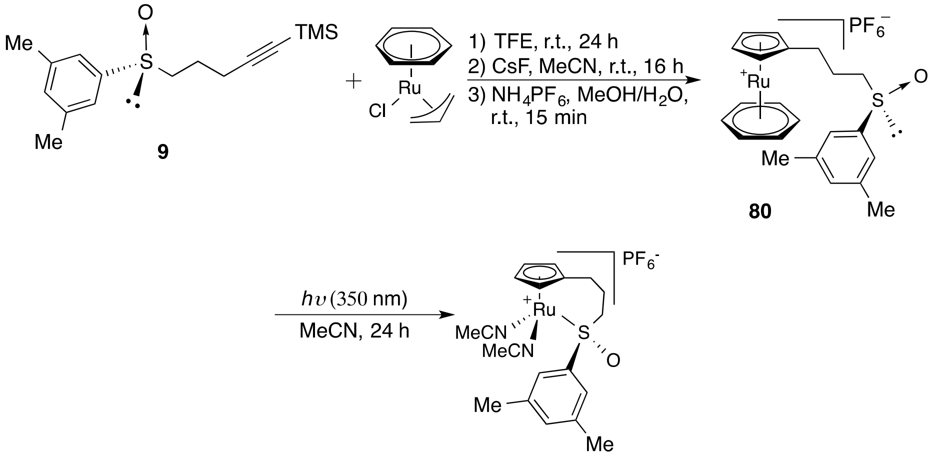

[(C_6H_6)Ru(allyl)Cl] (193 mg, 0.75 mmol, 1 equiv) was added to an oven-dried microwave vial with stir bar, fitted with a septum, and placed under argon. Alkyne **9** (243 mg, 0.83 mmol, 1.1 equiv) was added to the vial as a 0.28 M solution in 2,2,2-trifluoroethanol and the reaction was allowed to stir at room temperature for 24 hours. After 24 hours, the reaction was concentrated in vacuo and purified by acidic alumina chromatography (DCM to 2% MeOH in DCM to 5% MeOH in DCM) to yield a brown foamy powder (332 mg, 0.61 mmol, 81% yield) that was used directly in the next step.

The above ruthenium complex was dissolved in 2.44 mL of dry acetonitrile (0.25 M solution) and added to an oven-dried vial containing cesium fluoride (371 mg, 2.44 mmol, 4 equiv) placed under argon. The reaction was allowed to stir at room temperature for 16 hours before it was concentrated in vacuo and purified by acidic alumina chromatography (DCM to 2% MeOH in DCM to 5% MeOH in DCM) to yield a light-brown foamy solid (147 mg, 0.31 mmol, 51% yield) that was used directly in the ion exchange.

The above desilylated ruthenium complex was dissolved in 3.1 mL of a 1:1 v/v mixture of water/methanol. Ammonium hexafluorophosphate (303 mg, 1.86 mmol, 6 equiv) was added in one portion and the reaction was allowed to stir at room temperature for 15 minutes. 3 mL of DCM was added, and the layers were shaken and separated. The organic phase was washed twice with DCM (2 × 3 mL). The combined organics were dried over magnesium sulfate, filtered, and concentrated to yield **80** as a greenish-white foamy solid (168 mg, 0.29 mmol, 94% yield). $[\alpha]_D^{25}$= 49.49° (1.0, MeCN); IR (thin film) ν_{max}= 3077, 2923, 2855, 1633, 1605, 1443, 1103, 1044, 993, 853, 687; ^{1}H NMR (400 MHz, CD_3CN) δ 7.24 (s, 2H), 7.20 (s, 1H), 6.04 (s, 6H), 5.32 (dbr, 2H), 5.23 (b, 2H), 2.92-2.85 (m, 1H), 2.76-2.69 (m, 1H), 2.46-2.32 (m, 8H), 1.89-1.82 (m, 1H), 1.72-1.62 (m, 1H); ^{13}C NMR (CD_3CN) δ 140.34, 133.40, 122.30, 104.47, 87.18, 81.35, 80.57, 55.98, 27.05, 23.44, 21.32; ^{19}F NMR (CD_3CN) δ -73.02 (d, *J*= 751 Hz).

Mixed sandwich complex **80** was dissolved in 145 mL of acetonitrile (0.002 M solution) and added to large test tubes. The test tubes were fitted with septa and wrapped with electrical tape. The complex solutions were sparged with argon before being placed into a Rayonet Photochemical Reactor (equipped with F8T5-BL blacklight lamps, irradiating at 350 nm) and irradiated for 24 h. After 24 hours, the reaction was concentrated in vacuo to yield **19** as a foamy orange solid (132 mg, 0.23 mmol, 78% yield). $[\alpha]_D^{25}$= 56.12° (1.0, CH_3CN); IR (thin film) ν_{max}= 2925, 2259, 1605, 1441, 1187, 1113, 1079, 947, 824, 741, 690, 648, 556, 512, 497; ^{1}H NMR (400 MHz, CD_3CN) δ 7.36 (s, 2H), 7.16 (1H), 5.10 (m, 2H), 4.49 (brm, 1H), 4.28 (brm, 1H), 3.20-3.09 (m, 2H), 2.50-2.43 (m, 8H), 2.36 (s, 3H), 2.24-2.12 (m, 2H), 2.09 (s, 3H); ^{13}C-NMR (101 MHz, CD_3CN): δ 143.54, 139.34, 133.14, 123.25, 94.82, 82.07, 81.86, 67.91, 67.11, 58.85, 23.42, 21.45, 20.63; ^{19}F-NMR (376 MHz, CD_3CN): δ -73.04; LR-MS (m/z): [M -2MeCN -PF_6^-] calcd for $C_{16}H_{19}ORuS$: 361.0; found: 361.3.

(*R*)-(5-(Cyclohexylsulfinyl)pent-1-yn-1-yl)trimethylsilane (10)

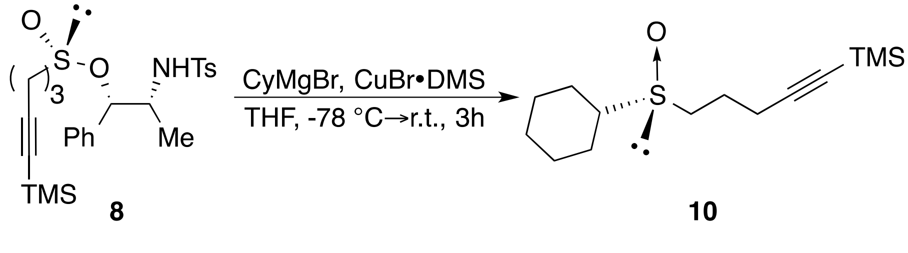

8 10

A 25 mL pear-shaped flask with stir bar was flame-dried and charged with copper (I) bromide-dimethyl sulfide complex (267 mg, 1.3 mmol, 5 equiv) and placed under argon. 1 mL of THF was added and the suspension was cooled to −78 °C. While the suspension sirred at −78 °C, 1.46 mL of a 1.62 M solution of cyclohexylmagnesium bromide in THF (2.34 mmol, 9 equiv) was added dropwise via syringe. The reaction mixture turned a bronze color while stirring at −78 °C for 30 minutes. After 30 minutes, 1 mL of a 0.2 M solution of **8** in THF (128 mg, 0.26 mmol, 1 equiv) was added dropwise via syringe. The reaction stirred for 1 hour at −78 °C and warmed to room temperature where it stirred for two hours. After two hours, the reaction was quenched with 2 mL of sat'd. aq. NH_4Cl and diluted with 2 mL of EtOAc. The layers were separated and the aqueous phase was washed two times with EtOAc (4 mL each). The combined organics were dried over $MgSO_4$, filtered, and concentrated in vacuo. Crude reaction mixture was purified *via* silica gel chromatography (1:1 hexanes/EtOAc). Sulfoxide **10** obtained as a pale yellow oil (48 mg, 0.18 mmol, 70% yield). $[\alpha]_D^{25}$= 14.12° (1.0, $CHCl_3$); IR (thin film) ν_{max}= 2958, 2855, 2174, 1586, 1451, 1378, 1345, 1248, 1044, 842, 759, 638; ^1H NMR (400 MHz, $CDCl_3$) δ 2.86-2.78 (m, 1H), 2.73-2.68 (m, 1H), 2.56 (tt, *J*= 11.2, 4 Hz, 1H), 2.46-2.37 (m, 2H), 2.12 (br, 1H), 2.03-1.85 (m, 5H), 1.70 (br, 1H), 1.51-1.21 (m, 5H), 0.13 (s, 9H); ^{13}C NMR ($CDCl_3$) δ 105.35, 86.41, 59.15, 47.69, 26.47, 25.69, 25.57, 25.32, 25.11, 21.85, 19.23, 0.22; LR-MS (m/z): [M + Na^+] calcd for $C_{14}H_{26}NaOSSi$: 293.1; found: 293.4.

Cyclohexyl sulfoxide catalyst (21)

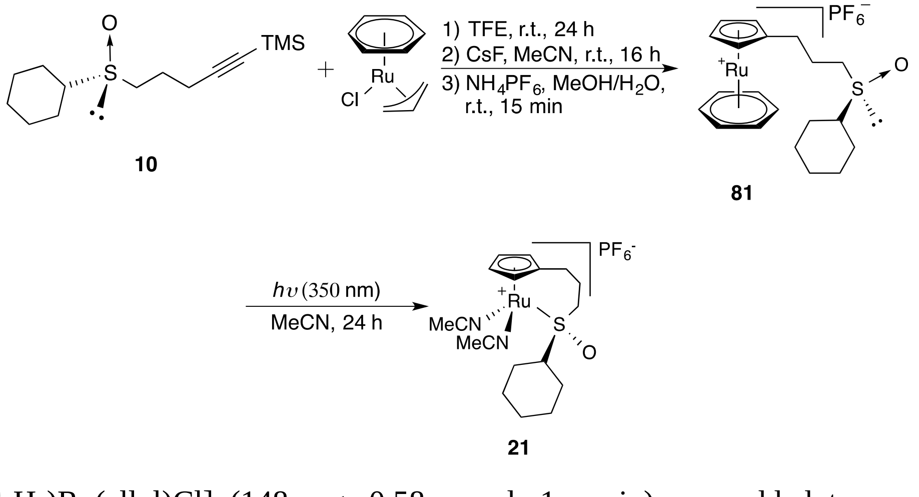

[$(C_6H_6)Ru(allyl)Cl$] (148 mg, 0.58 mmol, 1 equiv) was added to an oven-dried microwave vial with stir bar, fitted with a septum, and placed under argon. Sulfoxide **10**

(158 mg, 0.58 mmol, 1 equiv) was added to the vial as a 0.28 M solution in 2,2,2-trifluoroethanol and the reaction was allowed to stir at room temperature for 24 hours. After 24 hours, the reaction was concentrated in vacuo and purified by acidic alumina chromatography (DCM to 2% MeOH in DCM to 5% MeOH in DCM) to yield a brown foamy powder (259 mg, 0.49 mmol, 84% yield) that was used directly in the next step.

The above ruthenium complex was dissolved in 2 mL of dry acetonitrile (0.25 M solution) and added to an oven-dried vial containing cesium fluoride (595 mg, 3.92 mmol, 8 equiv) placed under argon. The reaction was allowed to stir at room temperature for 16 hours before it was concentrated in vacuo and purified by acidic alumina chromatography (DCM to 2% MeOH in DCM to 5% MeOH in DCM) to yield a light green foamy solid (115 mg, 0.25 mmol, 51% yield) that was used directly in the ion exchange.

The above desilylated ruthenium complex was dissolved in 2.5 mL of a 1:1 v/v mixture of water/methanol. Ammonium hexafluorophosphate (245 mg, 1.5 mmol, 6 equiv) was added in one portion and the reaction was allowed to stir at room temperature for 15 minutes. DCM (3 mL) was added, and the layers were shaken and separated. The organic phase was washed twice with DCM (2 × 3 mL). The combined organics were dried over magnesium sulfate, filtered, and concentrated to yield **81** as a greenish-white foamy solid (120 mg, 0.21 mmol, 84% yield). $[\alpha]_D^{25}$= 23.91° (1.0, $CHCl_3$); IR (thin film) ν_{max}= 3099, 2929, 2856, 1638, 1445, 1403, 1035, 922, 834, 752; ^{1}H NMR (400 MHz, $CDCl_3$) δ 6.15 (s, 6H), 5.41 (br, 2H), 5.32 (br, 2H), 2.70 (t, *J*= 7.2 Hz, 2H), 2.59-2.46 (m, 3H), 2.10-1.78 (m, 6H), 1.69 (d, *J*= 12 Hz, 1H), 1.50-1.20 (m, 6H); ^{13}C NMR ($CDCl_3$) δ 86.28, 80.69, 80.44, 80.03, 59.25, 47.40, 26.31, 26.24, 25.45, 25.27, 25.05, 24.84, 23.58; ^{19}F NMR ($CDCl_3$) δ -72.60 (d, *J*= 757 Hz).

Complex **81** was dissolved in 105 mL of acetonitrile (0.002 M solution) and added to large test tubes. The test tubes were fitted with septa and wrapped with electrical tape. The complex solutions were sparged with argon before being placed into a Rayonet Photochemical Reactor (equipped with F8T5-BL blacklight lamps, irradiating at 350 nm) and irradiated for 24 h. After 24 hours, the reaction was concentrated in vacuo to yield **21** as a foamy orange solid (127 mg, 0.15 mmol, 73% yield). $[\alpha]_D^{25}$= 12.11° (1.0, CH_3CN); IR (thin film) ν_{max}= 2935, 2858, 2258, 1647, 1455, 1403, 1194, 1070, 1004, 828, 741, 669, 649, 557; ^{1}H NMR (400 MHz, CD_3CN) δ 5.02 (br, 1H), 4.79 (brm, 1H), 4.25 (br, 1H), 4.19 (brm,

1H), 3.27(dd, J= 12.8, 4.4 Hz, 1H), 3.08 (tt, J= 11.6, 3.2 Hz, 1H), 2.67 (t, J= 11.6 Hz, 2H), 2.53 (m, 1H), 2.48 (s, 3H), 2.41 (s, 3H), 2.24-2.04 (m, 5H), 2.00-1.82 (m, 3H), 1.80-1.13 (m, 6H); ^{13}C-NMR (101 MHz, CD_3CN): δ 91.18, 87.58, 77.36, 71.27, 66.89, 64.46, 49.90, 25.76, 25.67, 25.63, 25.47, 25.21, 23.62, 20.94; ^{19}F-NMR (376 MHz, CD_3CN): δ -73.20; LR-MS (m/z): [M -2MeCN -PF_6^-] calcd for $C_{14}H_{21}ORuS$: 339.0; found: 339.3.

(1*S*,2*R*)-2-((4-Methylphenyl)sulfonamido)-1-phenylpropyl-(*S*)-2-methoxybenzenesulfinate (11)

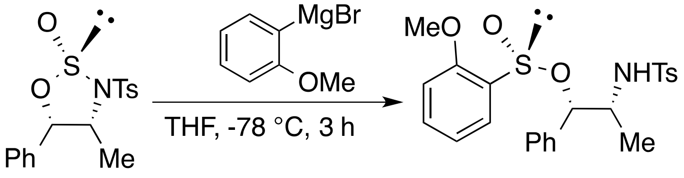

7 **11**

A 25 mL pear-shaped flask with stir bar was flame dried, charged with (S)-N-tosyl-methyl-phenyl-1,2,3-oxathiazolidine-2-oxide **7** (703 mg, 2 mmol, 1 equiv), fitted with a septum, and placed under argon. THF (7.77 mL) was added *via* syringe to create a 0.22 M solution. The solution was cooled to −78 °C and 1.32 mL of a 1.59 M solution of 2-methoxyphenylmagnesium bromide in THF was added dropwise via syringe to the oxathiazolidine-2-oxide solution. The reaction was allowed to stir at −78 °C for 4 hours. After 4 hours, the reaction was quenched at −78 °C with sat’d aq. $NaHCO_3$ (5 mL). The reaction was diluted with 5 mL of EtOAc and allowed to warm to room temperature. The phases were separated and the aqueous phase was washed two times with EtOAc (10 mL ea.). The combined organic layers were dried over Na_2SO_4, filtered, and concentrated in vacuo. The crude reaction mixture was purified via silica gel chromatography (3:1 hexanes:EtOAc) to give **11** as a colorless oil (1.03 g, 2.1 mmol, 66% yield). $[\alpha]_D^{25}$= 152.43° (1.0, $CHCl_3$); IR (thin film) ν_{max}= 3253, 2940, 1589, 1495, 1477, 1435, 1452, 1380, 1333, 1305, 1275, 1243, 1184, 1159, 1118, 1090, 1058, 1039, 1019, 994, 940, 907, 854, 815, 796, 753, 727, 699, 663, 617, 575, 547; ^{1}H-NMR (400 MHz; $CDCl_3$): δ 7.88 (dd, J = 7.7, 1.6 Hz, 1H), 7.79 (d, J = 8.2 Hz, 2H), 7.59-7.54 (m, 1H), 7.31-7.25 (m, 5H), 7.18 (t, J = 7.2 Hz, 1H), 7.10 (d, J = 7.8 Hz, 2H), 6.97 (d, J = 8.2 Hz, 1H), 5.56 (d, J = 9.5 Hz, 1H), 5.09 (d, J = 2.3 Hz, 1H), 3.68 (s, 3H), 3.60 (ddd, J = 9.5, 6.9, 2.6 Hz, 1H), 2.42 (s, 3H), 0.90 (d, J = 6.9 Hz, 3H); ^{13}C-NMR (101 MHz, $CDCl_3$): δ 157.38, 143.57, 138.34, 137.87, 134.60, 132.18,

129.94, 128.43, 128.17, 127.43, 126.05, 125.75, 121.21, 111.63, 84.03, 55.68, 54.44, 21.82, 14.92; HR-MS (m/z): [M + Na^+] calcd for $C_{23}H_{25}NNaO_5S_2$: 482.1; found: 482.3.

(*S*)-(5-((2-Methoxyphenyl)sulfinyl)pent-1-yn-1-yl)trimethylsilane (12)

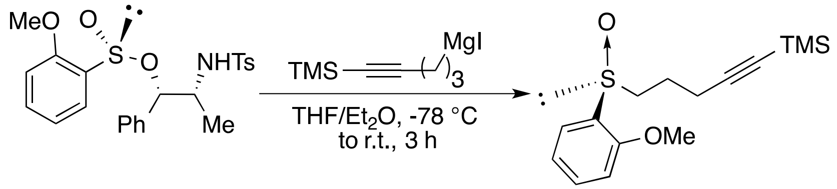

11 **12**

A 25 mL pear-shaped flask with stir bar was flame dried, fitted with a septum, and placed under argon. The flask was charged with a 5.42 mL of a 0.26 M solution of **11** (648 mg, 1.41 mmol, 1 equiv), which was added via syringe. The solution was cooled to −78 °C and 4.23 mL of a 2 M solution of (5-trimethylsilyl)-4-pentynlmagnesium iodide in Et_2O was added dropwise via syringe to the solution of **11** and the reaction was allowed to warm to r.t. where it stirred for 3 hours. After 3 hours, the reaction was quenched at −78 °C with sat'd aq. $NaHCO_3$ (5 mL). The reaction was diluted with 5 mL of EtOAc and allowed to warm to room temperature. The phases were separated and the aqueous phase was washed two times with EtOAc (10 mL ea.). The combined organic layers were dried over Na_2SO_4, filtered, and concentrated in vacuo. The crude reaction mixture was purified via silica gel chromatography (5:1:0.3 hexanes/EtOAc/MeOH) to give **12** as a colorless oil (354 mg, 1.2 mmol, 85% yield). $[\alpha]_D^{25}$= -202.25° (1.0, $CHCl_3$); IR (thin film) ν_{max}= 2958, 2840, 2173, 1586, 1477, 1436, 1307, 1272, 1249, 1069, 1039, 909, 843, 794, 758, 638; ^1H NMR (400 MHz, $CDCl_3$) δ 7.74 (d, *J*= 8 Hz, 1H), 7.42 (t, *J*= 8 Hz, 1H), 7.15 (t, *J*= 8 Hz, 1H), 6.89 (d, *J*= 8 Hz, 1H), 3.85 (s, 3H), 3.20-3.12 (m, 1H), 2.87-2.79 (m, 1H), 2.38-2.27(m, 2H), 2.07-1.99 (m, 1H), 1.78-1.71 (m, 1H), 0.10 (s, 9H); ^{13}C NMR ($CDCl_3$) δ154,84, 131.85, 130.66, 125.34, 121.45, 110.44, 105.36, 85.59, 55.60, 52.02, 20.91, 18.94, -0.01; LR-MS (m/z): [M + Na^+] calcd for $C_{15}H_{22}NaO_2SSi$: 317.1; found: 317.3.

o-Anisyl sulfoxide catalyst (20)

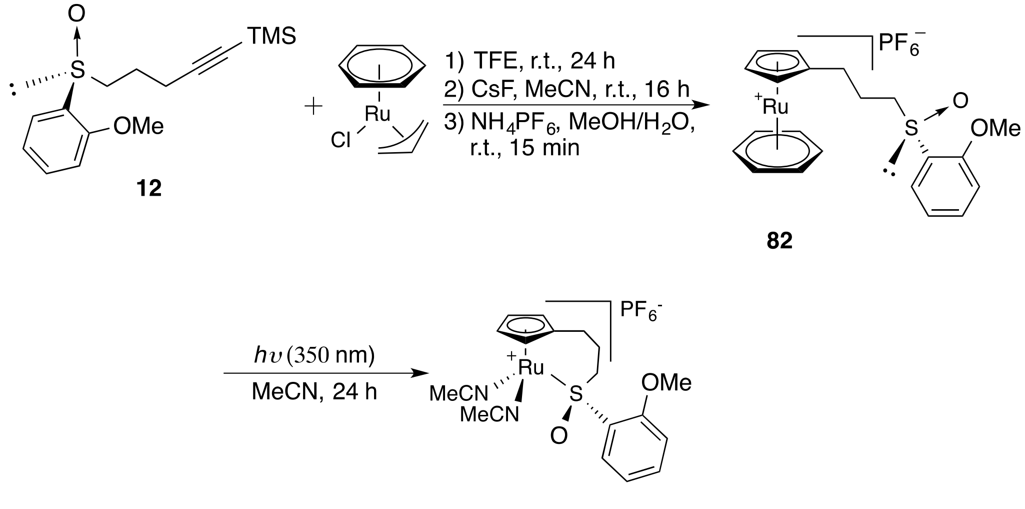

20

$[(C_6H_6)Ru(allyl)Cl]$ (279 mg, 1.1 mmol, 1 equiv) was added to an oven-dried microwave vial with stir bar, fitted with a septum, and placed under argon. Sulfoxide **12** (354 mg, 1.2 mmol, 1.1 equiv) was added to the vial as a 0.28 M solution in 2,2,2-trifluoroethanol (4.4 mL) and the reaction was allowed to stir at room temperature for 24 hours. After 24 hours, the reaction was concentrated in vacuo and purified by acidic alumina chromatography (DCM to 2% MeOH in DCM to 5% MeOH in DCM) to yield a brown foamy powder (525 mg, 0.96 mmol, 87% yield) that was used directly in the next step.

The above ruthenium complex was dissolved in 3.84 mL of dry acetonitrile (0.25 M solution) and added to an oven-dried vial containing cesium fluoride (583 mg, 3.84 mmol, 4 equiv) placed under argon. The reaction was allowed to stir at room temperature for 16 hours before it was concentrated in vacuo and purified by acidic alumina chromatography (DCM to 2% MeOH in DCM to 5% MeOH in DCM) to yield a light brown foamy solid (249 mg, 0.52 mmol, 54% yield) that was used directly in the ion exchange.

The above desilylated ruthenium complex was dissolved in 5.2 mL of a 1:1 v/v mixture of water/methanol. Ammonium hexafluorophosphate (512 mg, 3.14 mmol, 6 equiv) was added in one portion and the reaction was allowed to stir at room temperature for 15 minutes. 5 mL of DCM was added, and the layers were shaken and separated. The organic phase was washed twice with DCM (2 × 5 mL). The combined organic layers were dried over magnesium sulfate, filtered, and concentrated to yield **82** as a greenish-white foamy solid (286 mg, 0.49 mmol, 94% yield). $[\alpha]_D^{25}$= -119.97° (1.0, CH_3CN); IR (thin film) ν_{max}= 2919, 1633, 1584, 1470, 1435, 1272, 1238, 1033, 832; ^{1}H NMR (400 MHz, CD_3CN) δ 7.63

(d, *J*= 7.6 Hz, 1H), 7.50 (t, *J*= 7.6 Hz, 1H), 7.19 (t, *J*= 7.6 Hz, 1H), 7.04 (d, *J*= 8 Hz, 1H), 6.00 (s, 6H), 5.28 (br, 1H), 5.25 (s, 1H), 5.19 (br, 2H), 3.84 (s, 3H), 3.06-2.99 (m, 1H), 2.75-2.68 (m, 1H), 2.43-2.26 (m, 2H), 1.94-1.83 (m, 1H), 1.63-1.54 (m, 1H); ^{13}C NMR (CD_3CN) δ156.07, 133.21, 125.82, 122.24, 104.43, 87.16, 81.35, 80.55, 56.54, 52.34, 26.99, 23.28.; ^{19}F NMR (CD_3CN) δ -72.91 (d, *J*= 748 Hz).

Complex **82** (286 mg, 0.49 mmol) was dissolved in 245 mL of acetonitrile (0.002 M solution) and added to large test tubes. The test tubes were fitted with septa and wrapped with electrical tape. The complex solutions were sparged with argon before being placed into a Rayonet Photochemical Reactor (equipped with F8T5-BL blacklight lamps, irradiating at 350 nm) and irradiated for 24 h. After 24 hours, the reaction was concentrated in vacuo to yield **20** as a foamy orange solid (150 mg, 0.25 mmol, 52% yield). $[\alpha]_D^{25}$= -65.24° (1.0, CH_3CN); IR (thin film) ν_{max}= 2936, 2259, 1725, 1632, 1587, 1478, 1437, 1277, 1243, 1187, 1121, 1080, 1045, 1017, 830, 796, 761, 669, 648, 556, 533; ^{1}H NMR (400 MHz, CD_3CN) δ 7.79 (dd, *J*= 7.8, 1.6 Hz, 1H), 7.58 (dt, *J*= 8, 1.6 Hz, 1H), 7.24 (t, *J*= 7.2 Hz, 1H), 7.06 (d, *J*= 8.4 Hz, 1H), 5.12 (br, 1H), 5.04 (br, 1H), 4.22 (brm, 1H), 4.09 (br, 1H), 3.92 (s, 3H), 3.64 (dt,*J*= 12, 3.2 Hz,1H), 3.08 (dq,*J*= 16.8, 4 Hz, 1H), 2.68 (dq,*J*= 14.8, 3.2 Hz, 1H), 2.36 (s, 3H), 2.32-2.14 (m, 2H), 1.97-1.77 (m, 4H); ^{13}C-NMR (101 MHz, CD_3CN): δ 154.52, 133.70, 129.45, 126.96, 121.65, 111.64, 84.32, 80.81, 68.04, 68.02, 61.20, 55.65, 55.54, 23.48, 22.09; ^{19}F-NMR (376 MHz, CD_3CN): δ -73.15; LR-MS (m/z): [M -2MeCN - PF_6^-] calcd for $C_{14}H_{21}ORuS$: 363.0; found: 363.3.

1. Trost, B. M.; Ryan, M. C.; Rao, M.; Markovic, T. Z. *J. Am. Chem. Soc.* **2014**, *136* (50), 17422.

II. Spectral Data

The following pages contain ^{1}H and ^{13}C NMR data for the newly synthesized compounds described in the Supporting Information.

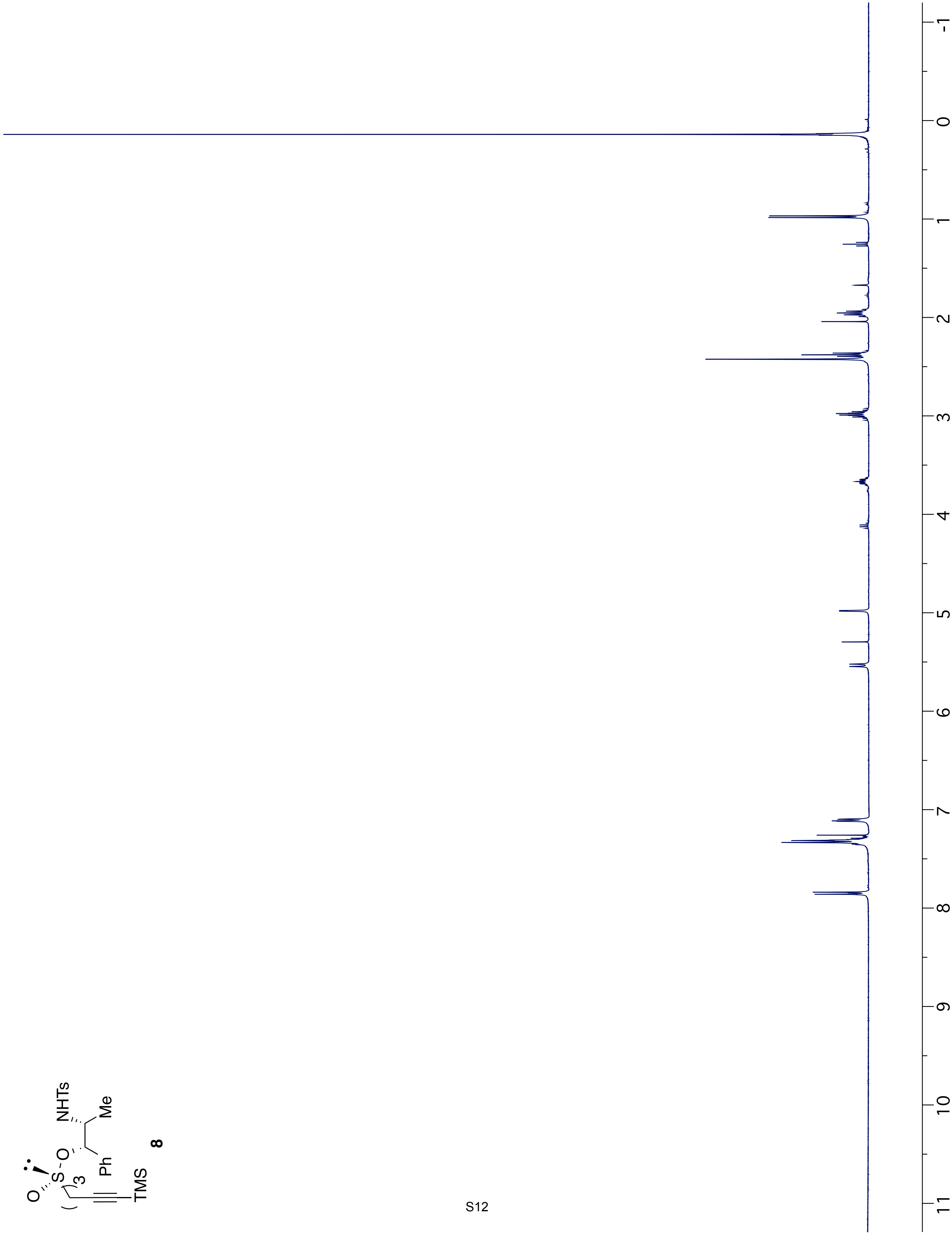
NHTs
Me
Ph
TMS
8
-1
0
1
2
3
4
5
6
7
8
9
10
11

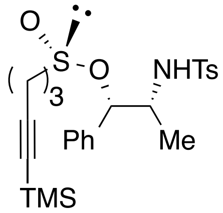

8

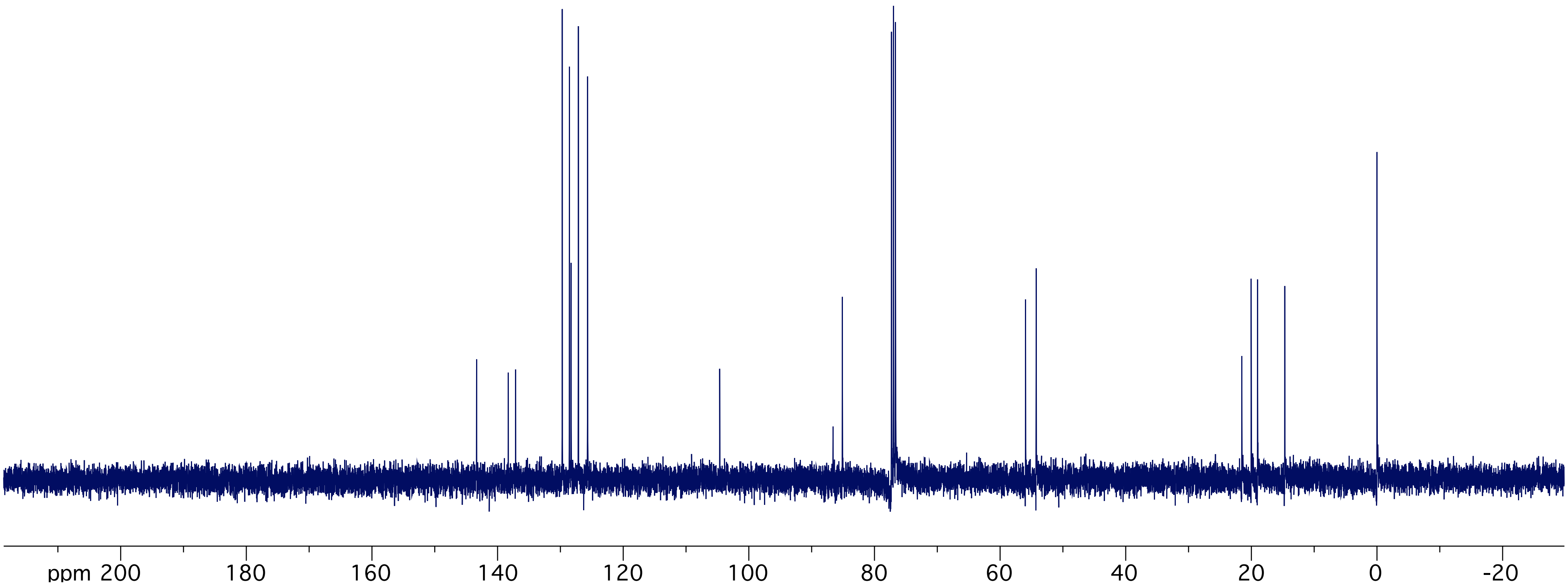

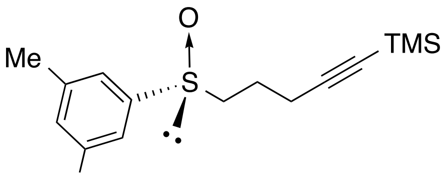

9

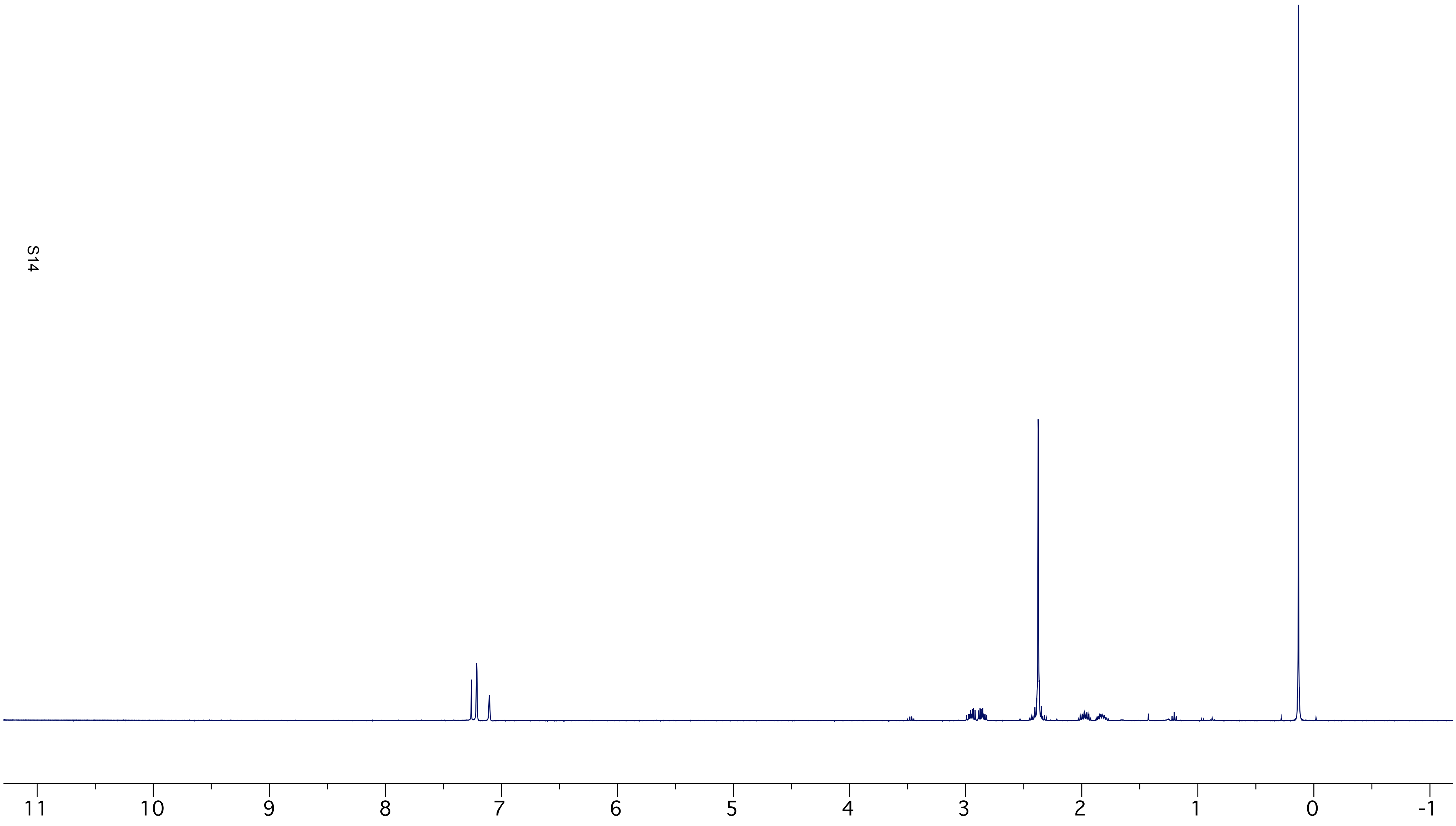

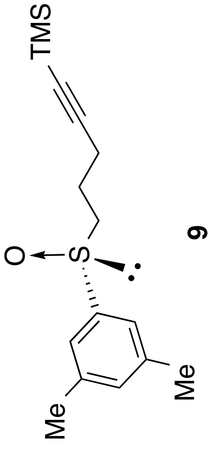

9

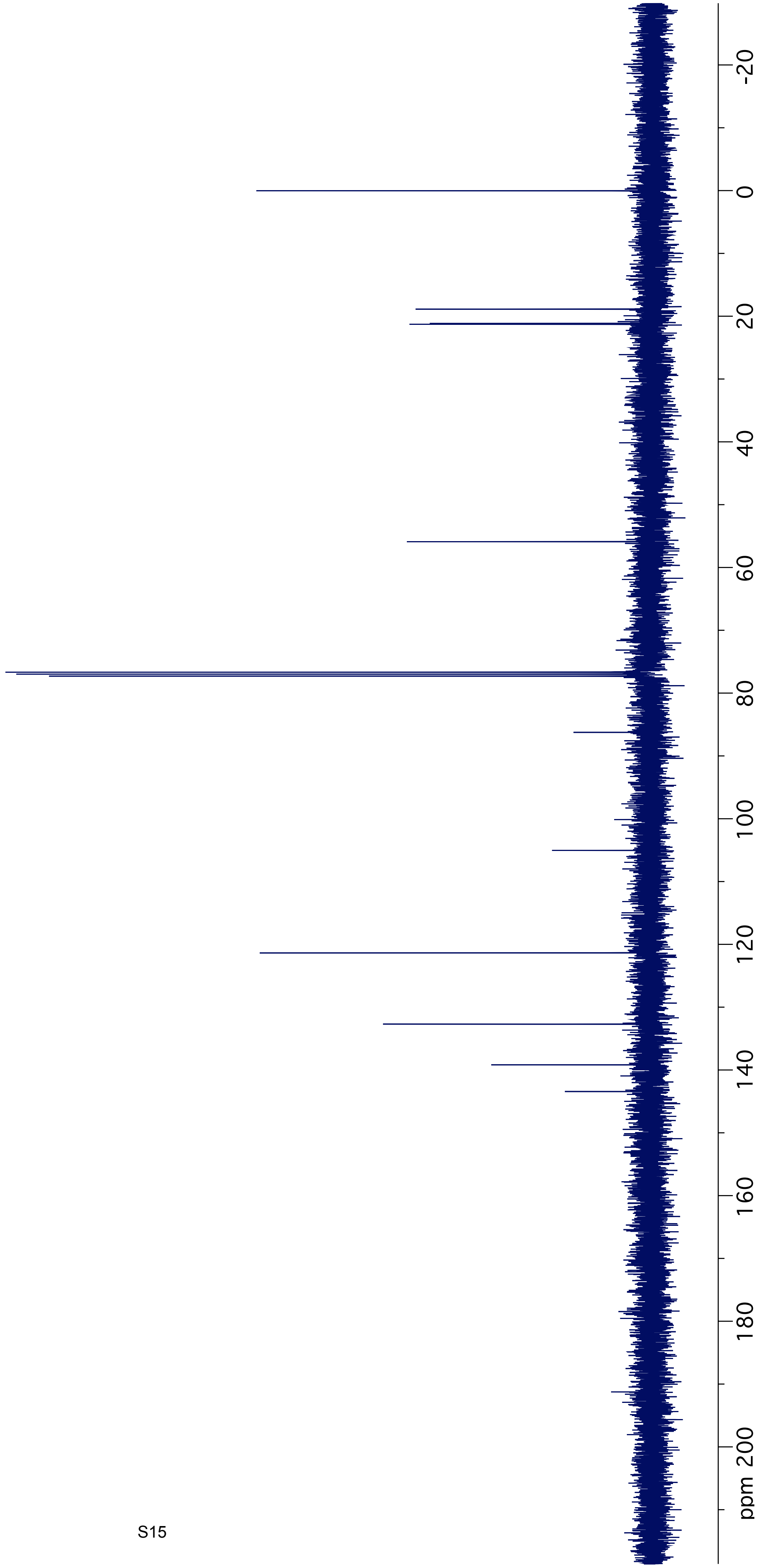

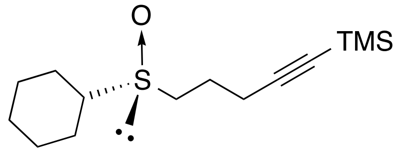

10

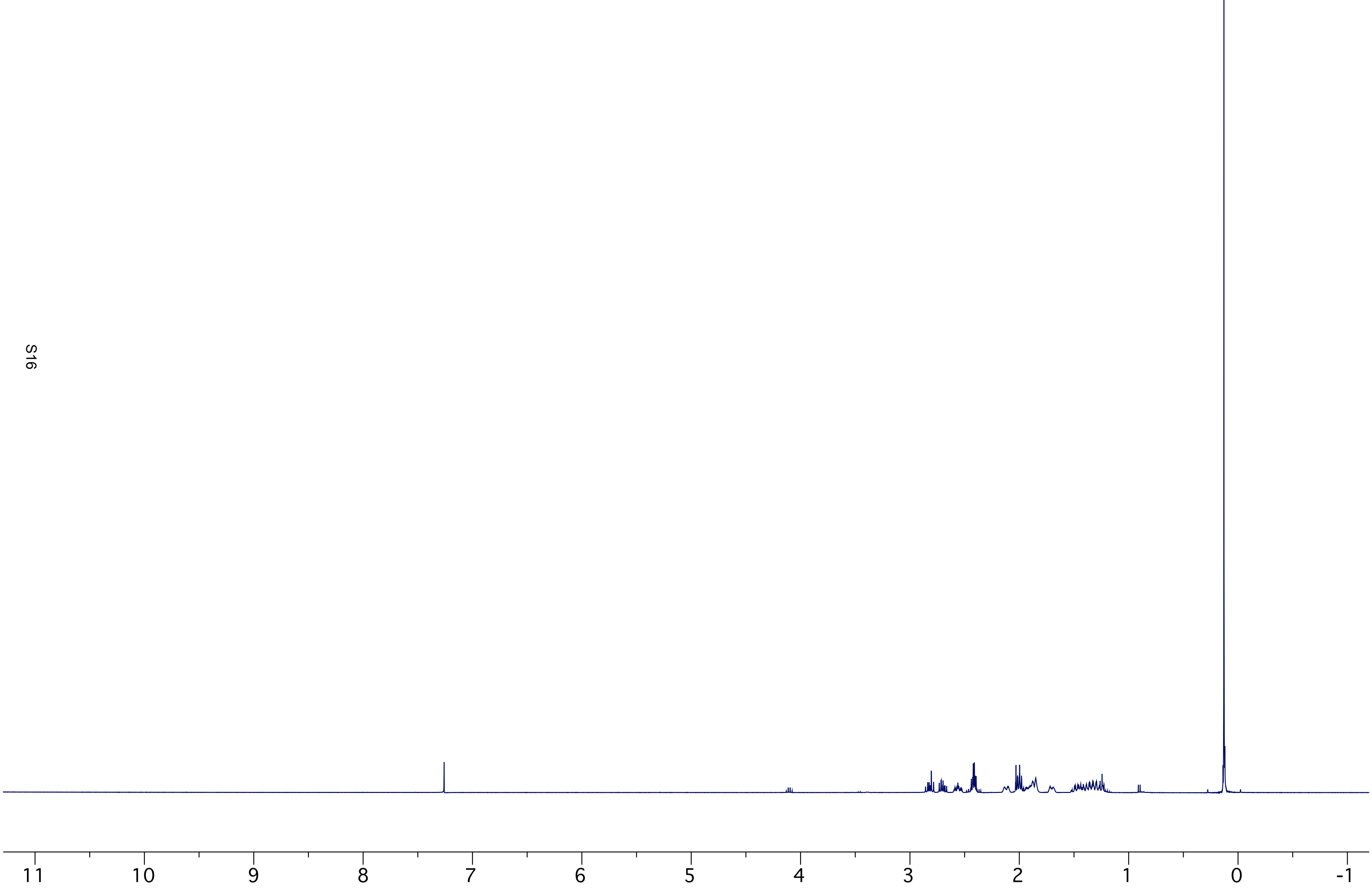

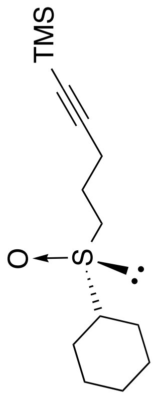

10

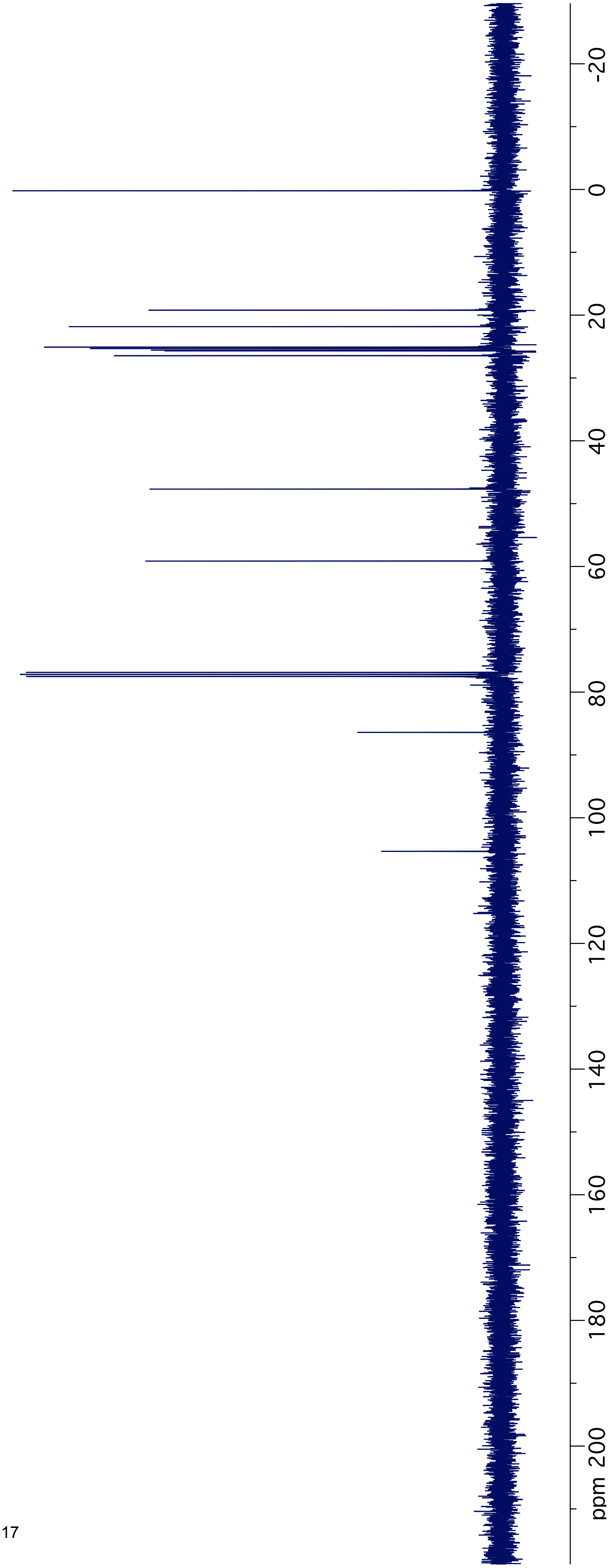

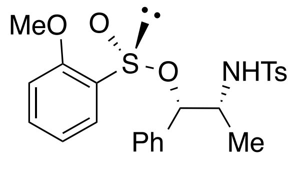

11

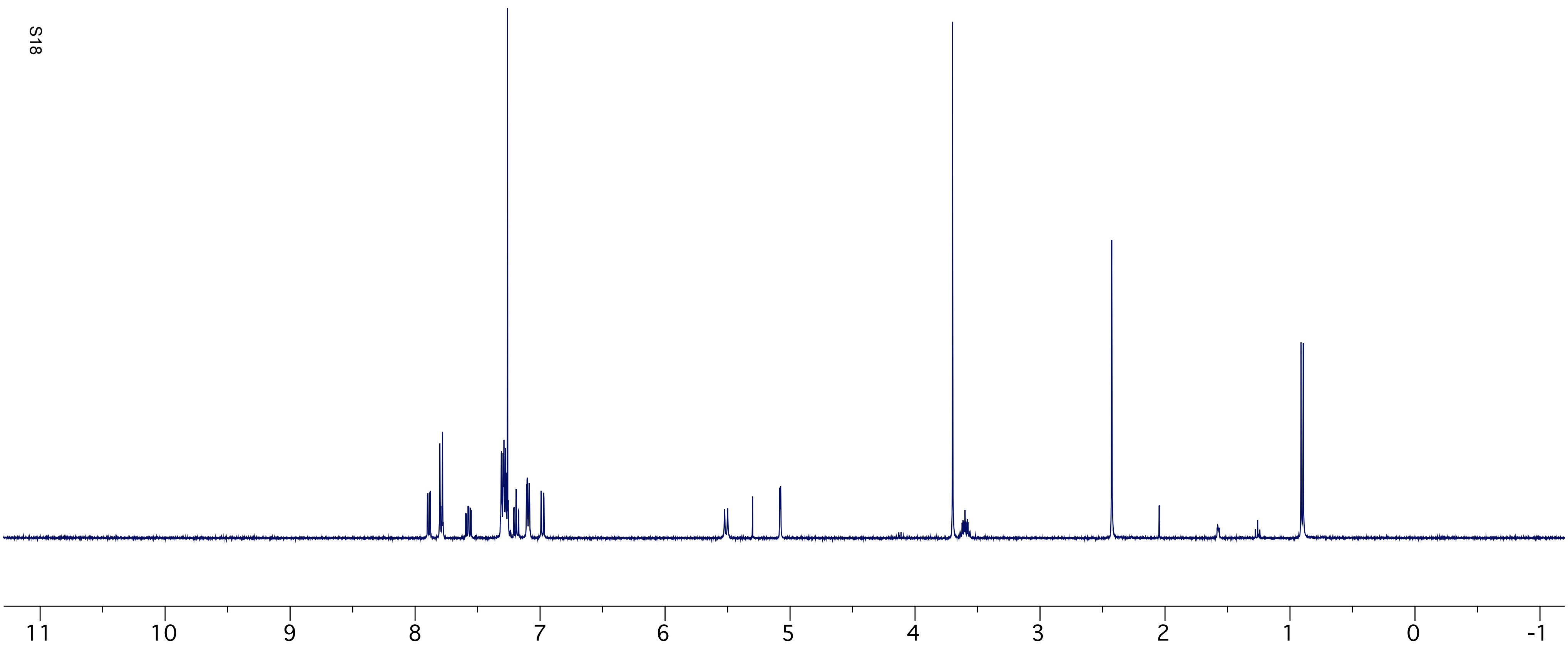

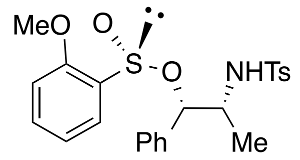

11

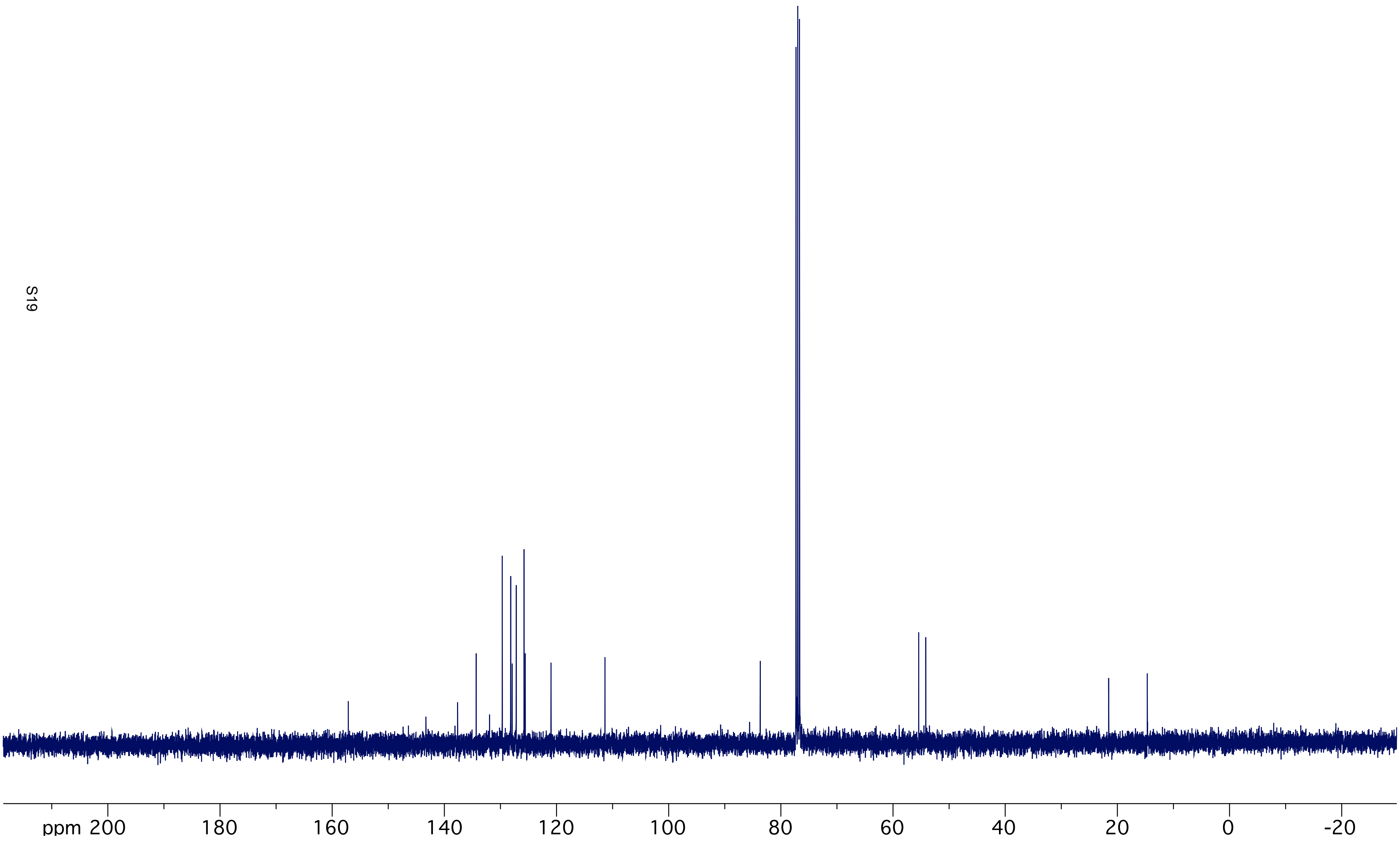

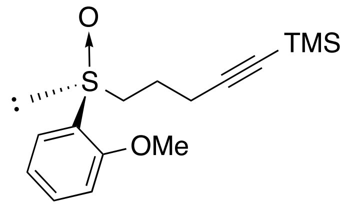

12

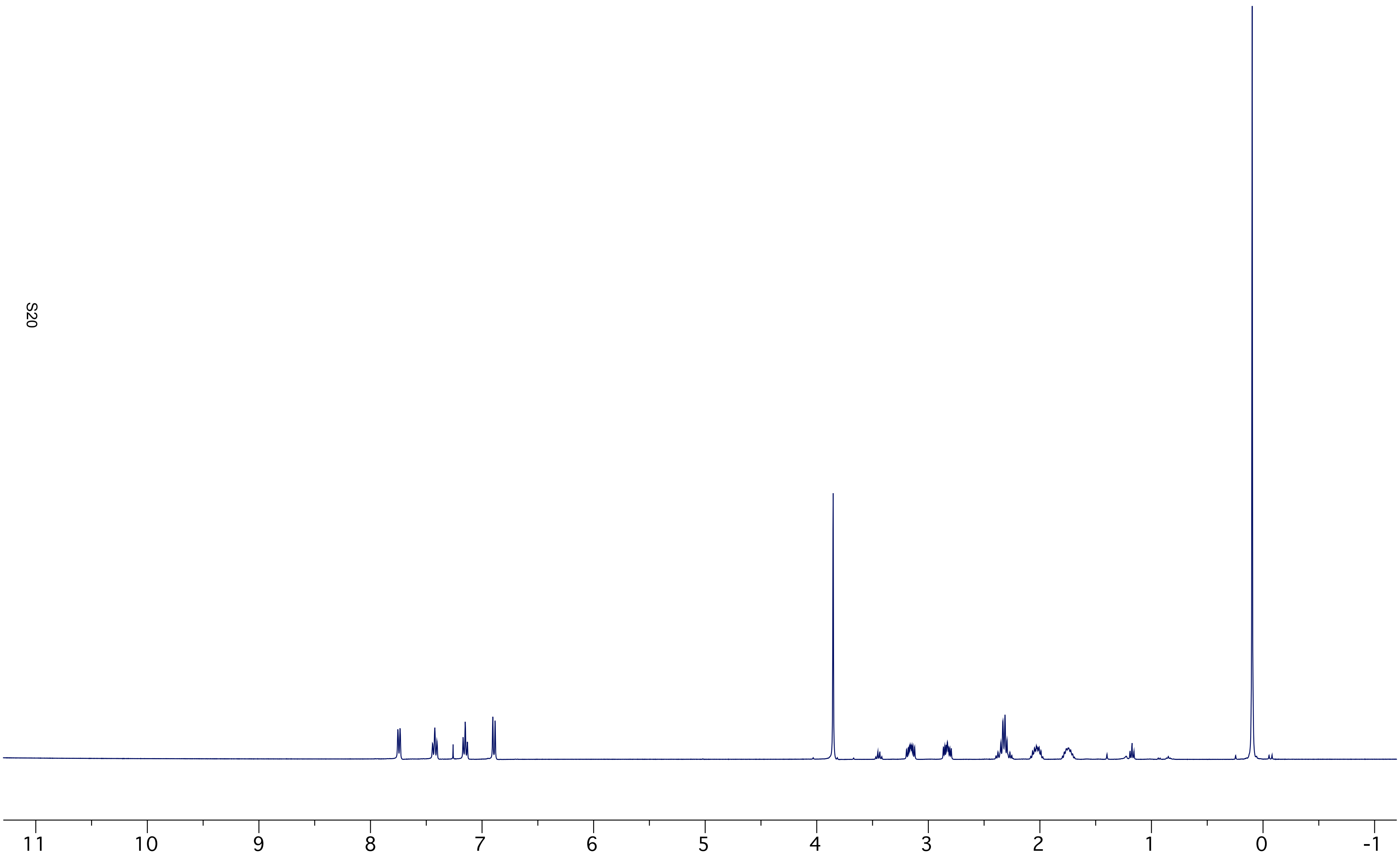

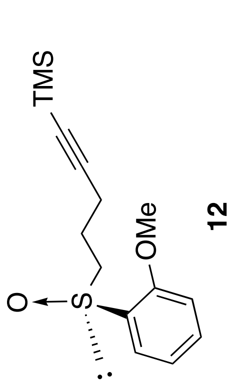

12

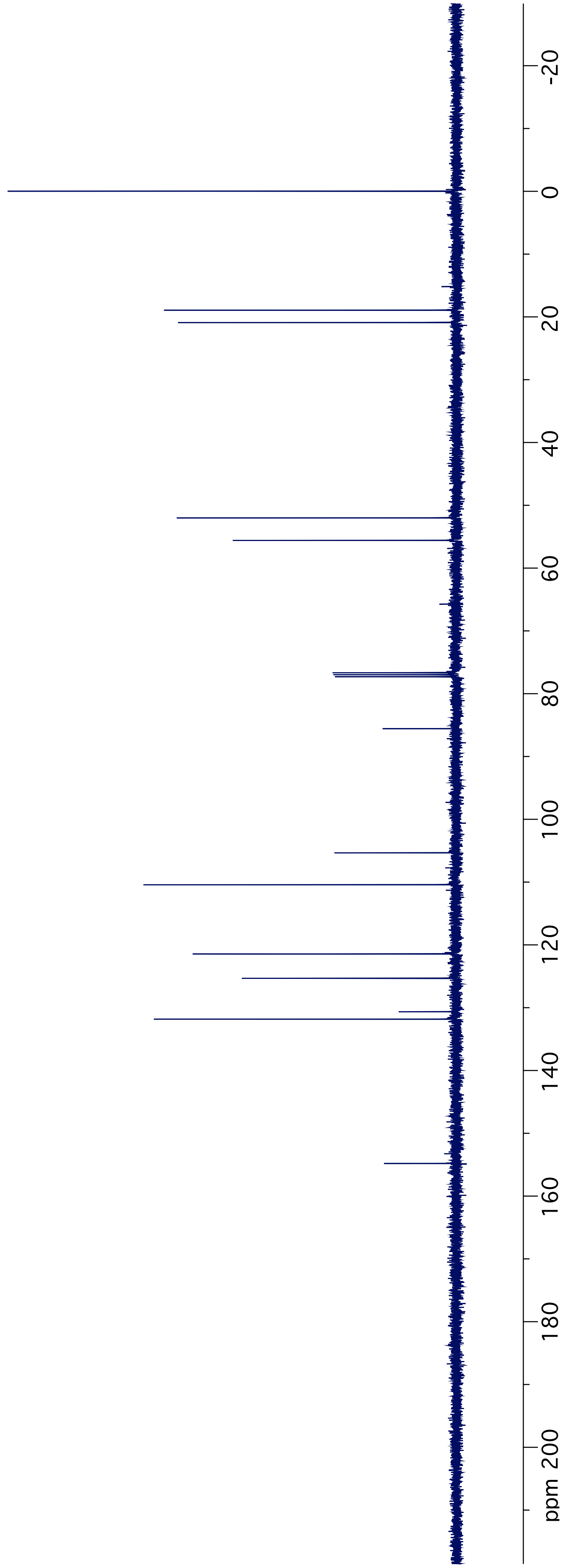

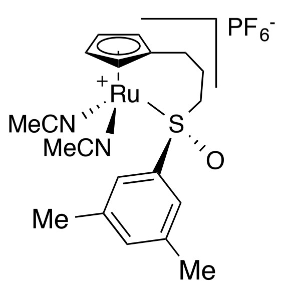

19

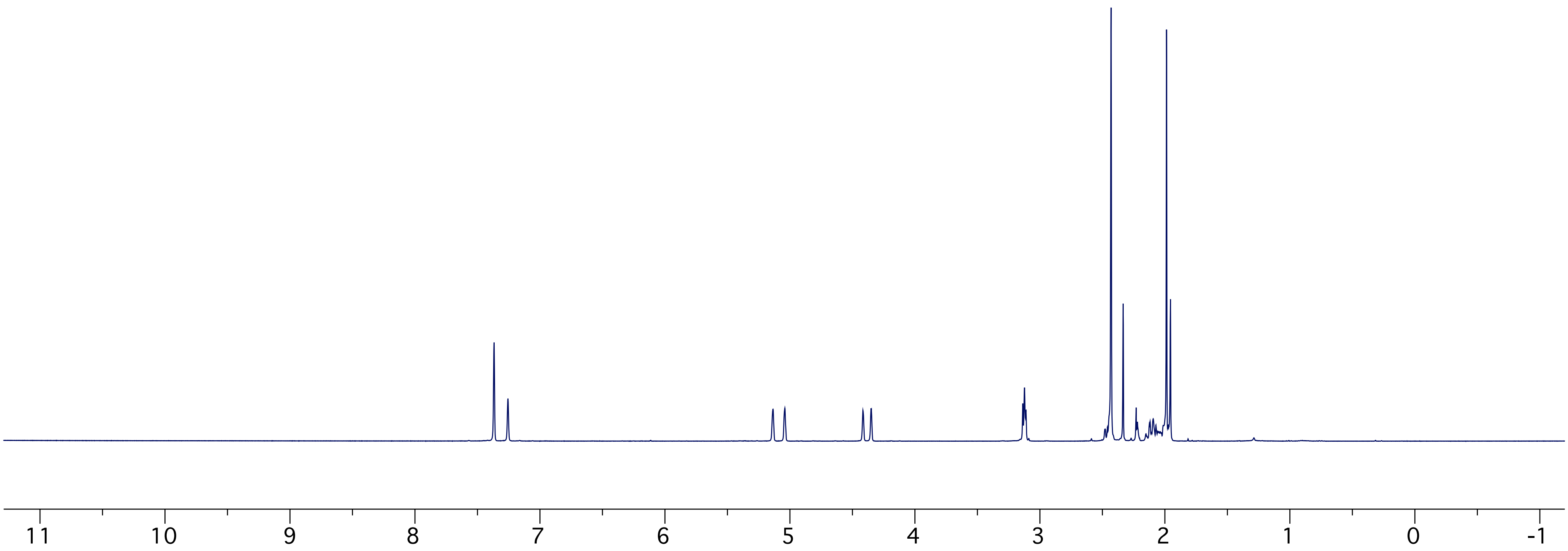

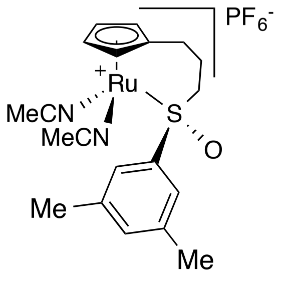
PF6-
Ru
MeCN
MeCN
S
O
Me
Me

19

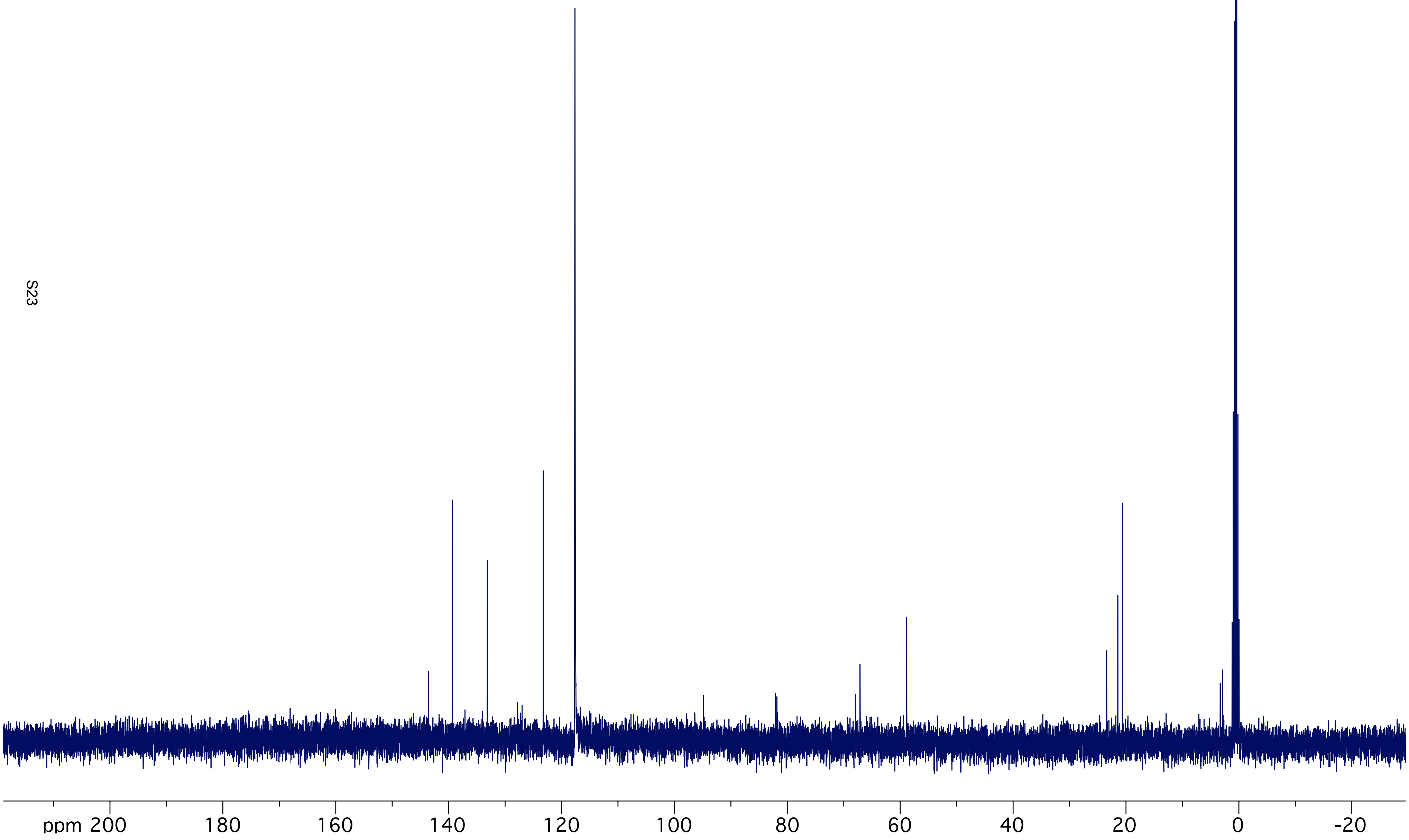
ppm 200
180
160
140
120
100
80
60
40
20
0
-20

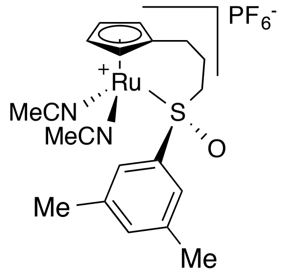

19

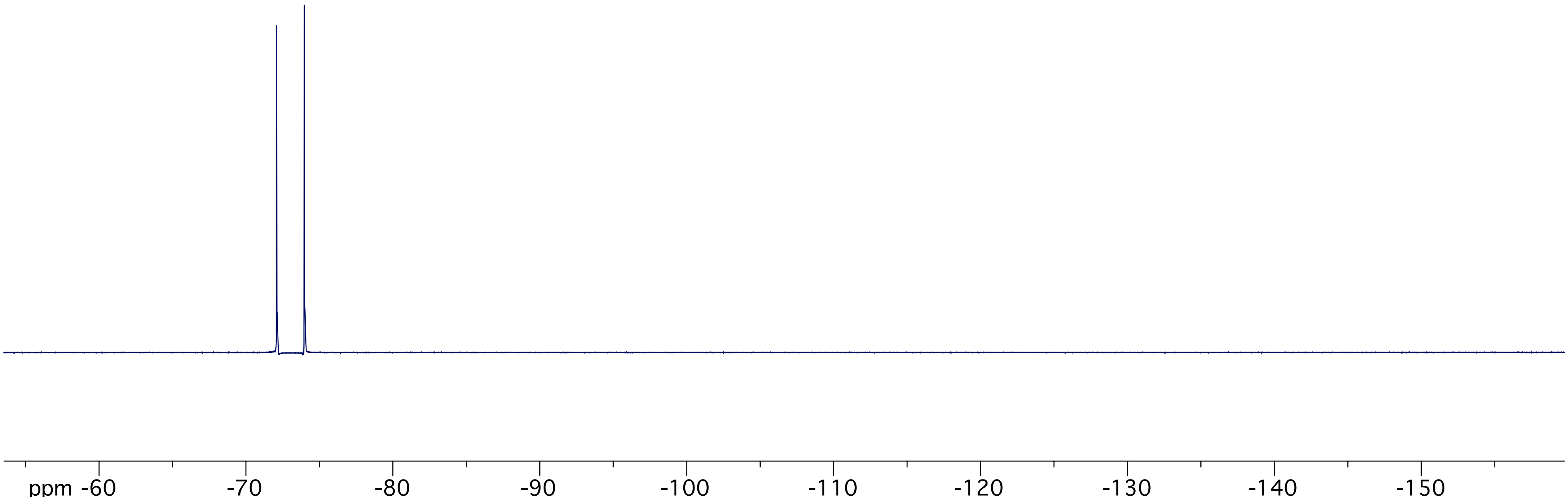

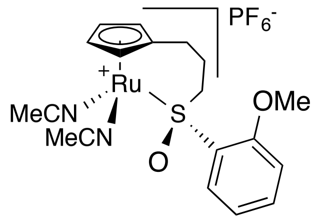

20

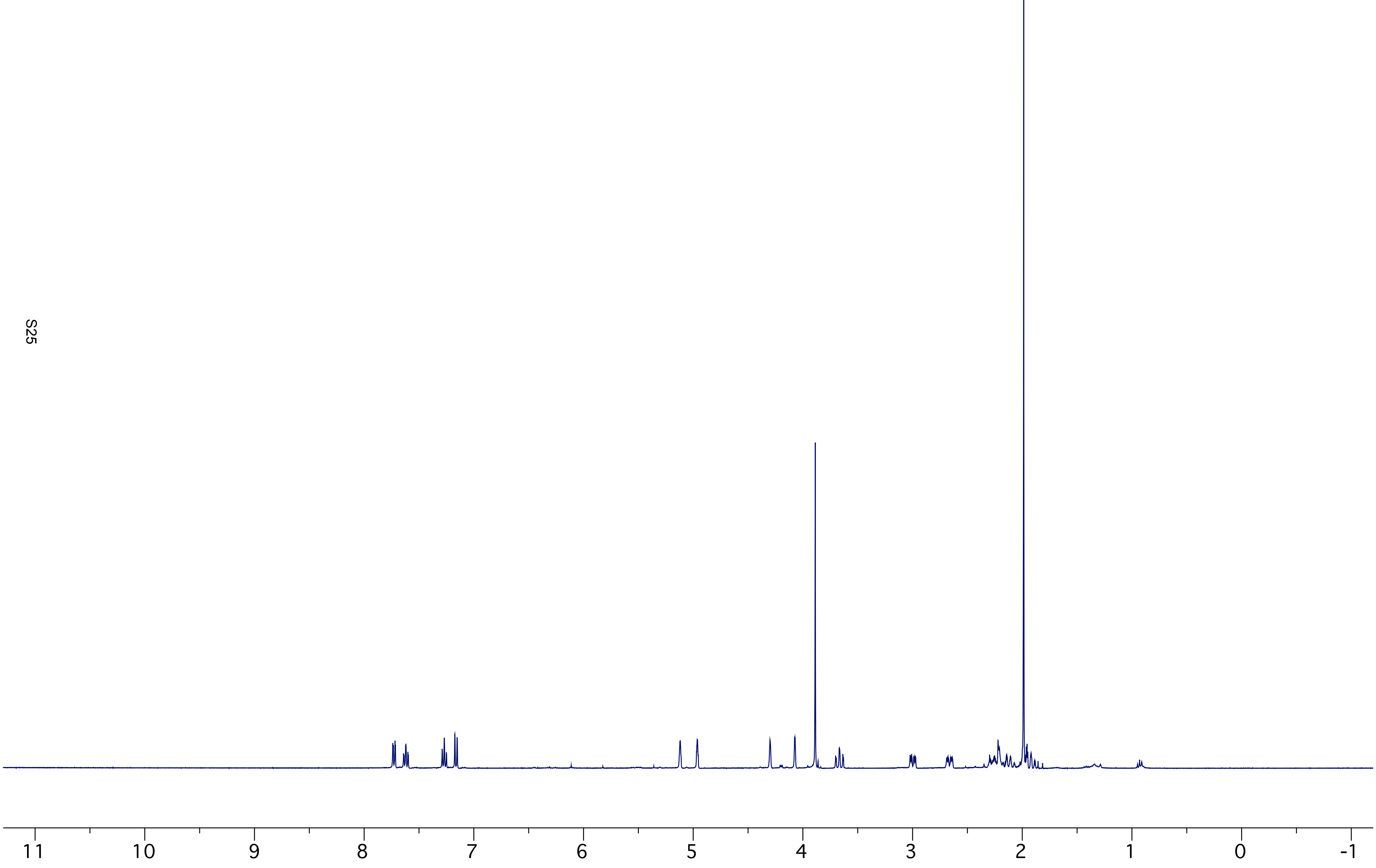

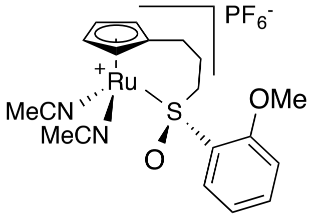
PF6-
Ru
MeCN
MeCN
S
O
OMe

20

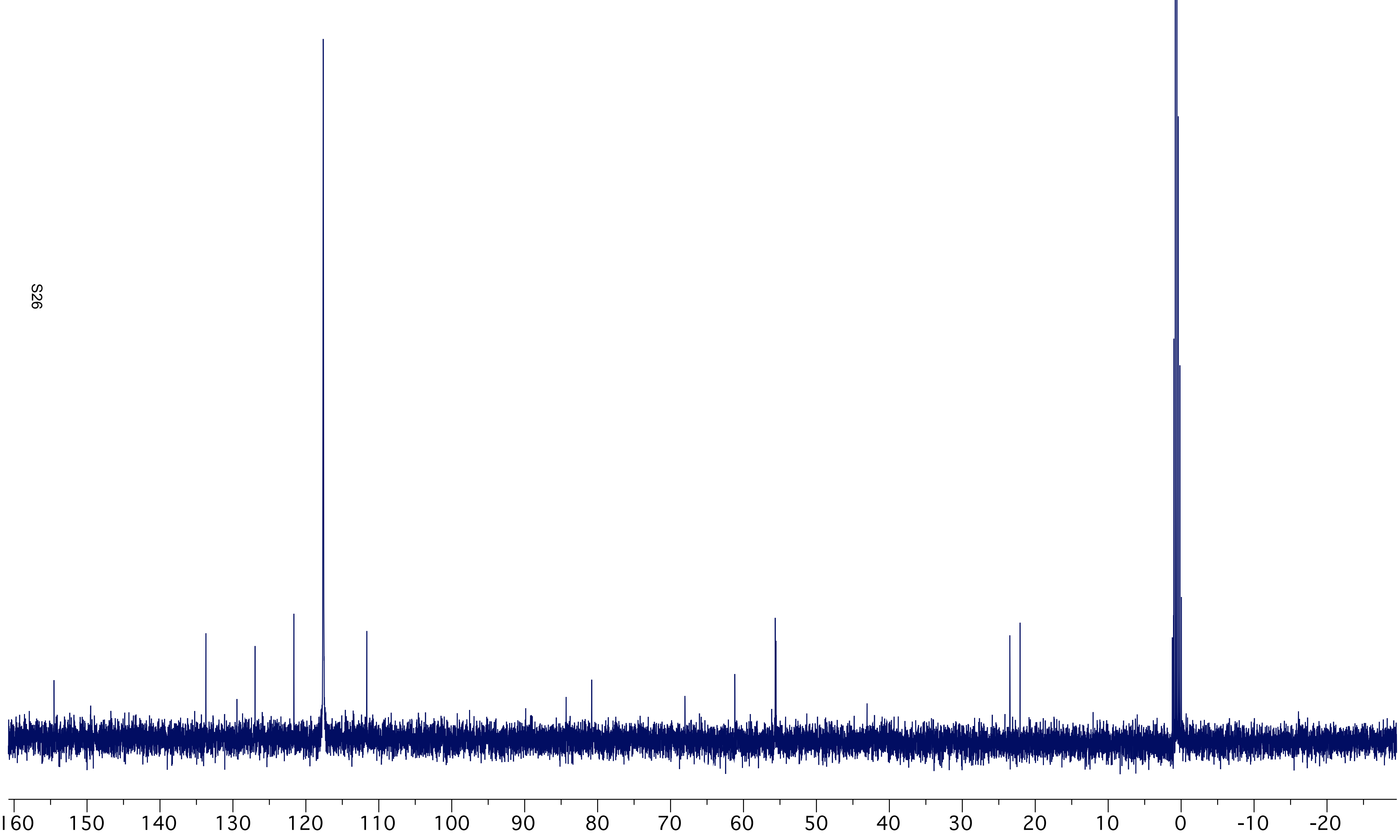
160
150
140
130
120
110
100
90
80
70
60
50
40
30
20
10
0
-10
-20

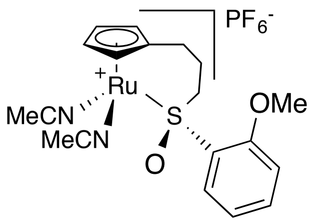

20

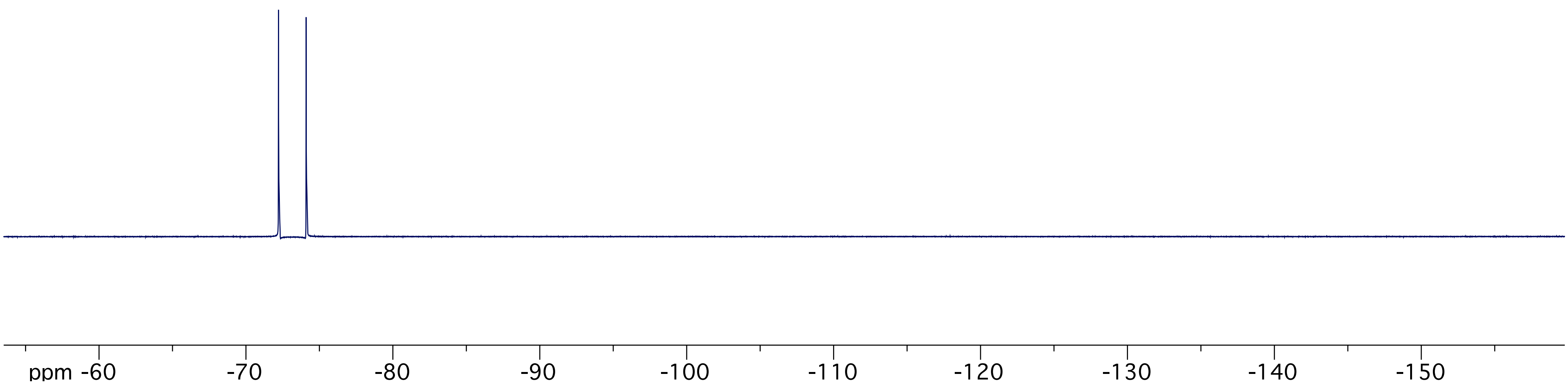

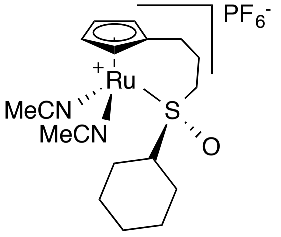

21

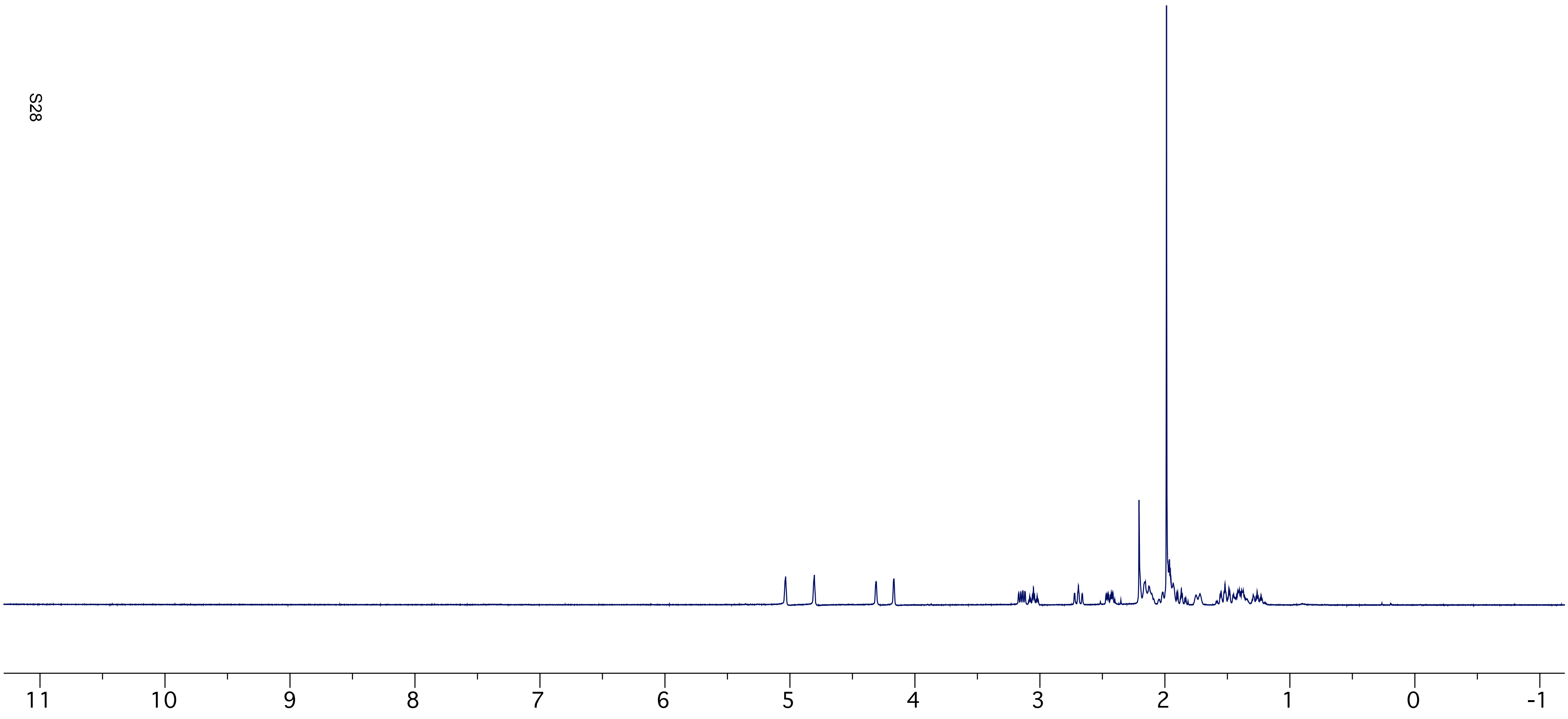

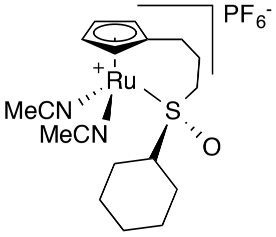

21

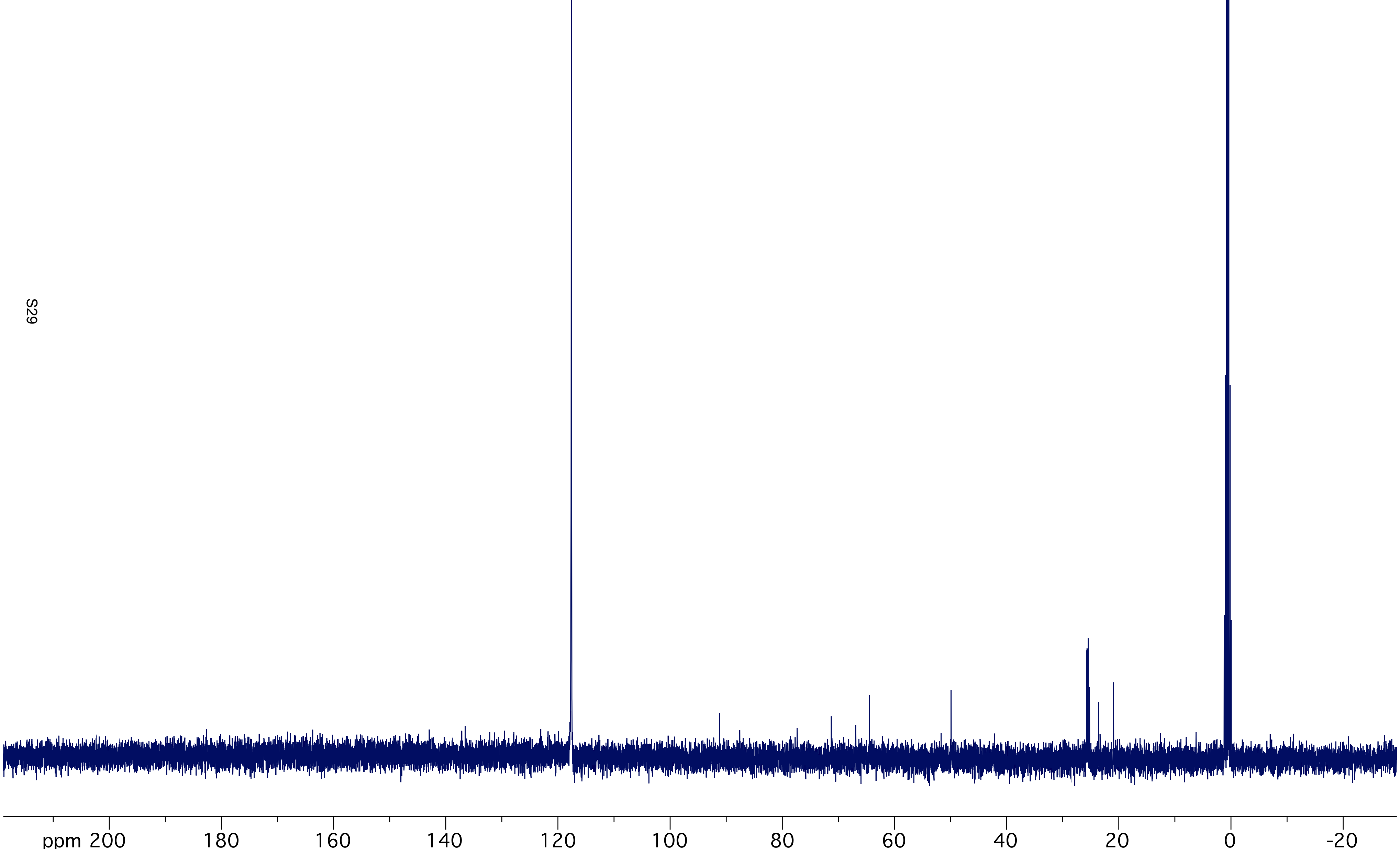

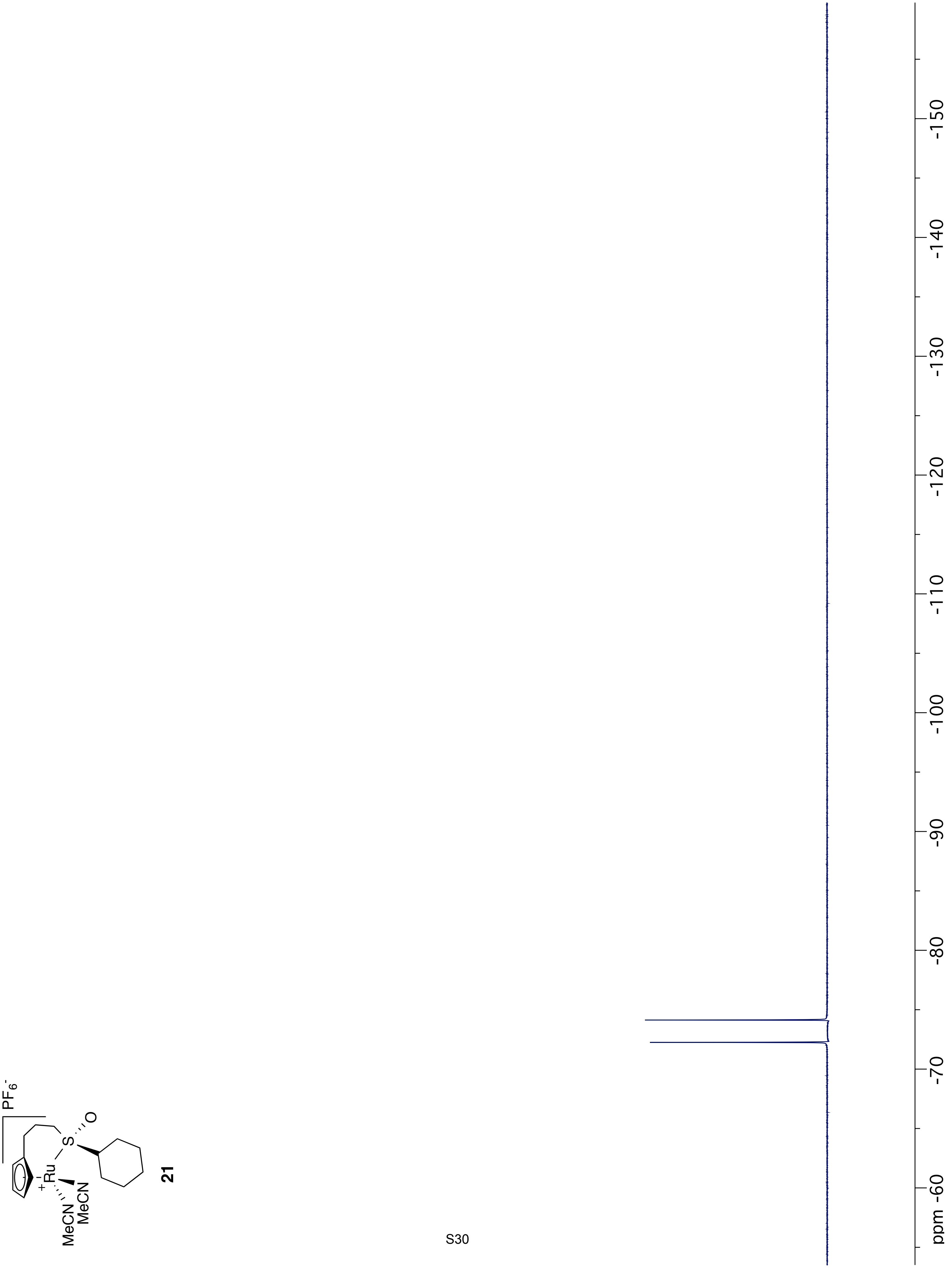

PF6-
Ru
S
O
MeCN
MeCN
21
ppm
-60
-70
-80
-90
-100
-110
-120
-130
-140
-150
